# Age‐associated metabolic and epigenetic barriers during direct reprogramming of mouse fibroblasts into induced cardiomyocytes

**DOI:** 10.1111/acel.14371

**Published:** 2024-11-14

**Authors:** Francisco Santos, Magda Correia, Rafaela Dias, Bárbara Bola, Roberta Noberini, Rita S. Ferreira, Diogo Trigo, Pedro Domingues, José Teixeira, Tiziana Bonaldi, Paulo J. Oliveira, Christian Bär, Bruno Bernardes de Jesus, Sandrina Nóbrega‐Pereira

**Affiliations:** ^1^ Department of Medical Sciences and Institute of Biomedicine – iBiMED University of Aveiro Aveiro Portugal; ^2^ Department of Experimental Oncology European Institute of Oncology (IEO), IRCCS Milan Italy; ^3^ Mass Spectrometry Center, Department of Chemistry University of Aveiro Aveiro Portugal; ^4^ LAQV/REQUIMTE University of Aveiro Aveiro Portugal; ^5^ CNC‐UC, Center for Neuroscience and Cell Biology University of Coimbra Coimbra Portugal; ^6^ CIBB, Center for Innovative Biomedicine and Biotechnology University of Coimbra Cantanhede Portugal; ^7^ Department of Oncology and Hematology‐Oncology University of Milano Milan Italy; ^8^ Institute of Molecular and Translational Therapeutic Strategies (IMTTS) Hannover Medical School (MHH) Hannover Germany; ^9^ Fraunhofer Institute for Toxicology and Experimental Medicine (ITEM) Hannover Germany

**Keywords:** aging, cardiomyocytes, direct reprogramming, epigenetic, fibroblasts, metabolism, mitochondria, regenerative biology

## Abstract

Heart disease is the leading cause of mortality in developed countries, and novel regenerative procedures are warranted. Direct cardiac conversion (DCC) of adult fibroblasts can create induced cardiomyocytes (iCMs) for gene and cell‐based heart therapy, and in addition to holding great promise, still lacks effectiveness as metabolic and age‐associated barriers remain elusive. Here, by employing MGT (Mef2c, Gata4, Tbx5) transduction of mouse embryonic fibroblasts (MEFs) and adult (dermal and cardiac) fibroblasts from animals of different ages, we provide evidence that the direct reprogramming of fibroblasts into iCMs decreases with age. Analyses of histone posttranslational modifications and ChIP‐qPCR revealed age‐dependent alterations in the epigenetic landscape of DCC. Moreover, DCC is accompanied by profound mitochondrial metabolic adaptations, including a lower abundance of anabolic metabolites, network remodeling, and reliance on mitochondrial respiration. In vitro metabolic modulation and dietary manipulation in vivo improve DCC efficiency and are accompanied by significant alterations in histone marks and mitochondrial homeostasis. Importantly, adult‐derived iCMs exhibit increased accumulation of oxidative stress in the mitochondria and activation of mitophagy or dietary lipids; they improve DCC and revert mitochondrial oxidative damage. Our study provides evidence that metaboloepigenetics plays a direct role in cell fate transitions driving DCC, highlighting the potential use of metabolic modulation to improve cardiac regenerative strategies.

## Graphical abstract

Age‐associated metabolic and epigenetic barriers during direct reprogramming of mouse fibroblasts into induced cardiomyocytes. Francisco Santos, Magda Correia, Rafaela Dias, Bárbara Bola, Roberta Noberini, Rita S. Ferreira, Diogo Trigo, Pedro Domingues, José Teixeira, Tiziana Bonaldi, Paulo J. Oliveira, Christian Bär, Bruno Bernardes de Jesus, and Sandrina Nóbrega‐Pereira*. Direct cardiac conversion (DCC) of fibroblasts into induced cardiomyocytes (iCMs) is a promising cardiac regenerative strategy but decreases with age. DCC is accompanied by alterations in the epigenetic landscape and mitochondria metabolism, network remodelling, and reliance in respiration (OxPhos). Metabolic modulations that decrease oxidative stress in the mitochondria (e.g., activation of mitophagy or dietary lipids) improve DCC in adult fibroblasts.

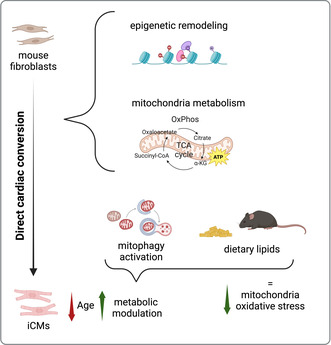

**Abbreviations**
Acta2: actin alpha 2, smooth muscle
Actc1: cardiac a‐actin
ACSS2: acyl‐CoA synthetase short chain family member 2
ANOVA: analysis of variance
ATAC‐seq: assay for transposase‐accessible chromatin using sequencing
Atp2a2: sarcoendoplasmic reticulum calcium ATPase
BSA: bovine serum albumin
Casq2: calsequestrin
CD: control diet
CFs: cardiac fibroblasts
ChIP: chromatin immunoprecipitation
ChIP‐seq: ChIP assays using sequencing
Col1a1: collagen, type I, alpha 1
Col3a1: collagen, type III, alpha 1
cTnT: cardiac troponin T
CMs: cardiomyocytes
CVD: cardiovascular disease
Cx43: connexin43
CQ: chloroquine
D day: post‐transduction
DCC: direct cardiac conversion
DEGs: differentially expressed genes
2‐DG: 2‐deoxy‐D‐glucose
Dox: doxycycline
DPBS: dulbecco's phosphate buffered saline
ECAR: extracellular acidification rate
ECM: extracellular matrix
eGFP: enhanced green fluorescent protein
Eln: elastin
FA: fatty acid
FACS: fluorescence‐activated cell sorting
FAO: fatty acid oxidation
Fap: fibroblast activation protein
FBS: fetal bovine serum
FC: flow cytometry
Fn1: fibronectin
GHMT: Gata4, Hand2, Mef2c,Tbx5
GMT: Gata4, Mef2c, Tbx5
GO: gene ontology
H3/4: histone 3/4
HBSS: hanks' balanced salt solution
HFD: high‐fat diet
ICC: immunocytochemistry
iCMs: induced cardiomyocytes
icMEFs: induced MGT construct mouse embryonic fibroblasts
IntDen: integrated density
iPSC: induced pluripotent stem cell
Jph2: junctophilin
α‐KG: α‐ketoglutarate
LC‐MS: liquid chromatography mass spectrometry
LFD: low‐fat/high‐fructose diet
LG: low glucose
LL: low lipids
MEFs: mouse embryonic fibroblasts
MFI: median fluorescence intensity
MGT: Mef2c, Gata4, Tbx5
α‐MHC: α‐myosin heavy chain
Myh6: a‐myosin heavy chain
Myl7: myosin, light polypeptide 7, regulatory
NAC: N‐acetylcysteine
NMCFs: neonatal mouse cardiac fibroblasts
NMCMs: neonatal mouse cardiomyocytes
OCR: oxygen consumption rate
PBS: phosphate buffered saline
PCA: principal component analysis
PFA: paraformaldehyde
PGC‐1α: peroxisome proliferator‐activated receptor gamma coactivator 1‐alpha
PI: propidium iodide
Pln: phospholamb
Postn: periostin
PTMs: posttranslational modifications
qPCR: quantitative polymerase chain reaction
RNA‐seq: RNA sequencing
ROS: reactive oxygen species
RT: reverse transcription
RVT: resveratrol
S: standard
SRB: sulforhodamine B
TCA: tricarboxylic acid cycle
TFs: transcription factors
TTFs: tail tip fibroblasts
TG: triglyceride
UroA: urolithin A
WB: western blotting

## 1 INTRODUCTION

Cardiovascular disease (CVD) is the leading cause of mortality in developed countries. CVD‐related pathologies are typically characterized by the irreversible loss of cardiomyocytes (CMs), which eventually leads to heart failure. Although conventional treatments exist, novel strategies are required to improve cardiac regeneration and patient welfare.

The use of cellular reprogramming and transdifferentiation in regenerative biology and age‐related pathologies is a promising area of research. Direct cardiac conversion (DCC) of resident cardiac or dermal fibroblasts by forced expression of cardiogenic transcription factors (TFs) in induced cardiomyocytes (iCMs) has emerged as an attractive strategy (Ieda et al., 2010; Qian et al., 2012; Song et al., 2012). Since its discovery, several strategies have been employed to improve DCC, including the manipulation of cardiogenic TFs, microRNAs, signaling pathways, and epigenetic factors (for a review, see (Wang et al., 2021; Kojima & Ieda, 2017)). For instance, the addition of Hand2 to a Gata4, Mef2c, and Tbx5 cocktail (GHMT) improved cardiac conversion efficiency (Song et al., 2012). Additionally, 5‐factor (GHMT + Nkx2.5) and 7‐factor (GMT + Myocd + Srf + Mesp1 + Smarcd3) cocktails further enhance cardiac reprogramming (Christoforou et al., 2013; Wada et al., 2013). Despite holding great promise, transdifferentiation of fibroblasts into iCMs is still not clinically effective, especially in aged cells (Hashimoto et al., 2018), owing to epigenetic restraints on somatic cell reprogramming. We previously identified a key molecular node that facilitates reprogramming of aged fibroblasts and safeguards stem cell pluripotency during aging (Bernardes de Jesus et al., 2018). Understanding the impact of systemic age‐associated barriers that limit cell fate conversion may offer new opportunities for cardiac regenerative strategies.

Following injury, the regeneration capacity of the adult mammalian heart is limited, whereas the neonatal heart is capable of substantial regeneration (Soonpaa et al., 1996). Interestingly, this is accompanied by a shift in the metabolic pathways and energetic fuels preferentially used by CMs, from embryonic glucose‐driven anaerobic glycolysis to the oxidation of substrates in the mitochondria in the adult stage (Lehman & Kelly, 2002; Lopaschuk et al., 1992). In addition to bioenergetics, metabolites are key regulators of epigenetic events and transcriptional programs that drive cell fate decisions (Intlekofer & Finley, 2019). In particular, FA‐depleted diets or the inhibition of FA oxidation (FAO) in vivo boost postnatal CM proliferation and extend the regenerative window in mice (Cardoso et al., 2020; Li et al., 2023). Glucose and lipid metabolism can greatly impact the intracellular pools of acetyl‐CoA and other metabolites, such as α‐ketoglutarate (α‐KG), that directly impact histone acetylation and methylation and epigenetic landscape transitions in skeletal muscle regeneration (Yucel et al., 2019).

Despite their importance, our understanding of the systemic metabolic and (age‐associated) epigenetic determinants of cell lineage conversion during the transdifferentiation of fibroblasts into iCMs remains elusive. As dyslipidemia is a risk factor for CVDs (Stone et al., 1974), defining the impact of systemic lipids on DCC and cardiac regeneration is a priority. Here, we used MGT transduction to generate iCMs from mouse fibroblasts at different ages and subjected them to differential nutritional stimuli to characterize the metabolic and epigenetic transitions taking place during transdifferentiation with age, allowing an unprecedented characterization of the molecular and metabolic circuitries limiting DCC and providing grounds for metabolic improvement of cardiac regeneration.

## 2 RESULTS

### 2.1 Direct reprogramming of fibroblasts into iCMs decreases with age

**FIGURE 1:**
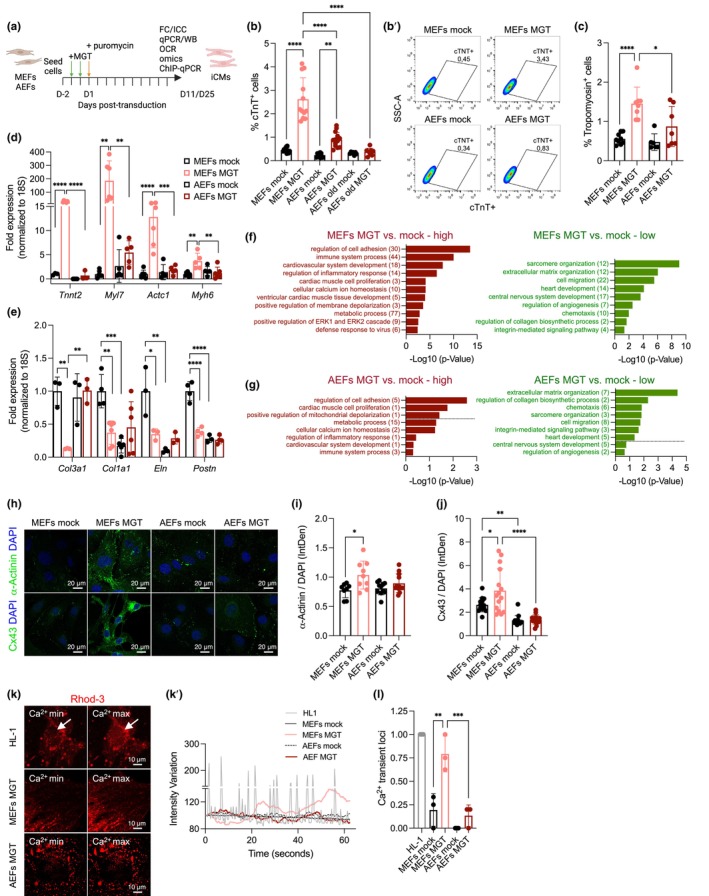
Direct reprogramming of fibroblasts into iCMs decreases with age. (a) Schematic representation of the procedure for direct cardiac conversion of fibroblasts into iCMs. Fibroblasts (mouse embryonic, MEFs; adult skin ear fibroblasts, AEFs) are seeded followed by two rounds of MGT retroviral infections (green arrow, days −1 and 0) and puromycin selection at day D1 post‐transduction (orange arrow). iCMs are analyzed at 11 or 25 days post‐transduction (D11/D25) by several analytical techniques. (b, c) Flow cytometry quantification of the percentage of cardiac troponin (cTnT) positive cells (b), representative plots (b') and expression of tropomyosin (c) in MEFs, AEFs (4–6 months) or AEFs old (12 months) mock or MGT retroviral‐infected on D11/12. (d, e) qPCR analysis of the relative expression of the indicated genes in MEFs and AEFs mock or MGT retroviral‐transduced on D11/12 (fold to MEFs mock). (f, g) Bulk RNA‐sequencing analysis in mock and MGT‐transduced MEFs (f) and AEFs (g) at D11 with GO terms of biological processes enriched in MEFs or AEFs MGT versus mock. Numbers in parenthesis indicate the gene number of each GO term. Left panel plot represents enriched functional categories for upregulated genes (red); right panel represents enriched functional categories for downregulated genes (green), where –log10 (0.05) = 13,010 and dash line indicates significancy limit. (h–j) Representative images (h) and quantification of immunofluorescence for α‐actinin (i) and Connexin 43 (Cx43) (j) per cell (integrated density, IntDen) at D11 in mock and MGT transduced MEFs and AEFs. (k, l) Calcium transients measured by Rhod‐3 dye labeling (k), intensity variation trace (k') and quantification of ratio of Ca^2+^ transient‐expressing loci per well (l) in mock and MGT‐transduced MEFs and AEFs at D25, and HL‐1 cells. Each point on the plot indicates individual measurements and mean ± s.d or ratio of cells per well (l) of *n* = 3–14 biological replicates from three independent or one representative experiment (b–g) or *n* = 8–17 cells (i, j, l) analyzed from one representative experiment. *p* values were calculated by one‐way ANOVA followed by post‐hoc Tukey's test. *p < 0.05; **p < 0.01; ***p < 0.001; ****p < 0.0001. Graphs were created using GraphPad Prism (RRID: SCR_002798) and flow cytometry plots (b') with FlowJo (RRID: SCR_008520), schema (a) with BioRender.com. Scale bars, 20 μm in (h) and 10 μm in (k).

DCC from skin fibroblasts is an attractive cell‐based cardiac regenerative strategy; however, it is ineffective. We used mouse embryonic fibroblasts (MEFs) and adult skin dermal ear‐derived fibroblasts (AEFs) from adult C57BL/6J mice from different ages (4–6 or 12 months old) for retrovirus‐induced transduction of the cardiogenic factors Mef2c, Gata4, and Tbx5 (hereafter referred to as MGT) (Wang et al., 2015) or empty backbone (mock), followed by puromycin selection (Figure 1a). Among the primary fibroblasts used for reprogramming, AEFs and old AEFs exhibit a general decrease in the expression of key markers of activated fibroblasts, such as *Postn* (periostin) and *Fap* (fibroblast activation protein) (compared to MEFs) (Figure S1a), whereas the expression of *Fn1* (fibronectin) was only reduced in AEFs and *Acta2* (myofibroblast gene; actin alpha 2, smooth muscle) expression was only reduced in old AEFs (Figure S1a).

We observed that, at 11 days post‐transduction (D11), MEFs MGT exhibit a higher percentage of cells expressing cardiac troponin T (cTnT) (Figure 1b,b', mean 2.62% vs. 0.90%) and tropomyosin (Figure 1c) (compared to AEFs MGT), highlighting the decreased generation of iCMs (MGT‐transduced cells expressing cardiomyocyte markers) with age. Moreover, MGT‐transduction of AEFs from older animals (12 months) resulted in an even lower percentage of cTNT+ cells (mean, 0.28%), which did not differ from that of mock‐infected cells (Figure 1b). Quantitative PCR (qPCR) analysis at D11 revealed an increased expression of reprogramming factors (*Mef2c*, *Gata4*, *Tbx5*) in both MGT‐transduced MEFs and AEFs (Figure S1b). However, induction of cardiac markers, such as the sarcomere structure genes *Tnnt2* (troponin T2, cardiac), *Myl7* (myosin, light polypeptide 7, regulatory), *Actc1* (cardiac α‐actin), and *Myh6* (α‐myosin heavy chain) (Figure 1d), muscle contractility and components of the excitation‐contraction coupling, such as *Pln* (phospholamb), *Casq2* (calsequestrin), *Jph2* (junctophilin), and *Atp2a2* (sarcoendoplasmic reticulum calcium ATPase, SERCA) (Figure S1c), and repression of fibroblast markers such as *Col3a1* (collagen, type III, alpha 1), *Col1a1* (collagen, type I, alpha 1), *Eln* (elastin), *and Postn* (Figure 1e) was only observed in MGT‐transduced MEFs with only minor changes in AEF‐transduced cells.

To investigate the global transcription profile, we performed bulk RNA sequencing (RNA‐seq) of MGT (or mock)‐transdifferentiated MEFs and AEFs on D11, along with the HL‐1 mouse cardiomyocyte cell line. According to the heatmap of the correlation coefficients, the biological replicates of HL‐1 cells and MGT‐ or mock‐transduced MEFs were highly correlated and grouped together (Figure S1d). Principal component analysis (PCA) (Figure S1e) showed clustering of samples into three groups (HL‐1, MEFs, and AEFs), with PC1 reflecting the differences between HL‐1 and fibroblasts (regardless of age or transduction) and PC2 accounting for the difference between embryonic and adult fibroblasts (regardless of transduction). Differentially expressed gene (DEG) analysis revealed that 1192 genes were differentially expressed between MEFs MGT and mock, and only 381 genes were differentially expressed between AEFs MGT and mock (Figure S1f), whereas a similar number of genes were differentially expressed between HL‐1 and MEFs or AEFs MGT (7488 and 7528, respectively, Figure S1f). Gene Ontology (GO) analysis further revealed that, compared to mock, upregulated genes in MGT‐transduced MEFs were enriched for GO terms for biological processes associated with cardiac function (cardiac muscle, inflammatory response, membrane depolarization, cellular calcium, metabolic processes) (Figure 1f, left and Table S1), while the downregulated genes were associated with fibroblast signatures (cell migration, chemotaxis, angiogenesis), ECM organization (collagen biosynthesis), and integrin‐mediated signaling (Figure 1f, right and Table S1). Some categories of MEFs MGT were suggestive of incomplete reprogramming into iCMs, for example, lower expression of genes related to sarcomere organization (Figure 1f, right), higher expression of cell adhesion (Figure 1f, left), and no significant suppression of mitotic cell cycle genes (*p* = 0.064, Table S1), whereas minor enrichment in epicardial, endothelial, or vascular gene signatures was observed in MEFs MGT when compared to the mock (Table S1). Importantly, MGT‐transduced adult fibroblasts exhibited fewer significant differences in the enrichment of GO terms associated with cardiac function (Figure 1g, left, and Table S1) and the downregulation of genes associated with fibroblast signatures (Figure 1g, right, and Table S1), highlighting the decreased efficiency of DCC in adult fibroblasts.

MGT‐transduced MEFs exhibit increased expression of the sarcomeric protein α‐actinin (Figure 1h,i), despite lacking well‐defined structures at this stage (D11), and higher expression of the gap junction protein Connexin43 (Cx43) at the cell–cell contacts (Figure 1h,j) in comparison with mock or MGT‐transduced AEFs. Next, we analyzed the intracellular Ca^2+^ flux by Rhod3 dye labeling after 4 weeks of culture (D25) and observed that MGT‐transduced MEFs exhibited spontaneous Ca^2+^ oscillation at a lower frequency than HL‐1 cardiomyocytes (Figure 1k,k', Movie S1 and S2) and an increased proportion of Ca^2+^ transient‐expressing loci compared to MGT‐transduced AEFs (Figure 1k,l, Movie S3), which only represent partially reprogrammed cells. Overall, these results suggested that MGT‐transduced MEFs express reprogramming characteristics at higher levels than adult skin fibroblasts, which is compatible with the early stages of cardiac reprogramming.

### 2.2 Epigenetic transitions during direct reprogramming of fibroblasts into iCMs differ with age

**FIGURE 2:**
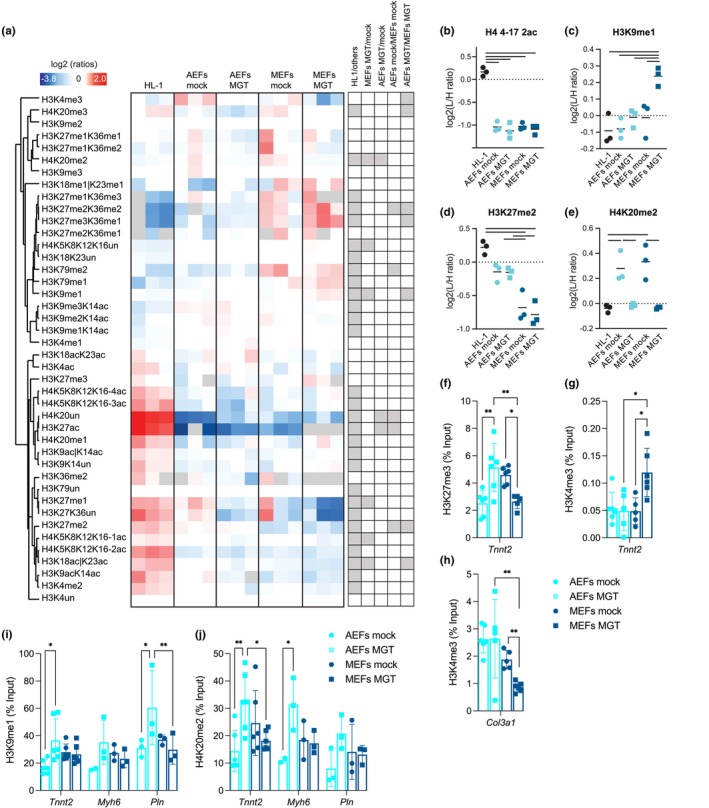
Epigenetic transitions during direct reprogramming of fibroblasts into iCMs differ with age. (a–e) Mass spectrometry‐based histone acetylation and methylation profiling on mock and MGT‐transduced MEFs and AEFs on D11, and HL‐1 mouse cardiomyocyte cell line. Heatmap display of histone PTM levels. Ratio of L/H (light/heavy) relative abundances was obtained using a spike‐in strategy (light channel: Sample, heavy channel: Spike‐in standard), and normalization over the average ratios across samples. Histone peptides were clustered based on Pearson's correlation, grey squares indicates peptides that were not quantified. The panel on the right shows significant changes (grey) for the indicated comparisons (a). Display of selected histone modified peptides showing levels of bi‐acetylated form of H4 lysine 4–17 (b), mono‐methylated form of H3 lysine 9 (c), di‐methylated form of H3 lysine 27 (d) and di‐methylated form of H4 lysine 20 (e), the bar indicates a *p* value <0.05. (f–j) ChIP‐qPCR for H3K27me3 (f), H3K4me3 (g, h), H3K9me1 (i) and H4K20me2 (j) for cardiac (*Tnnt2*, *Myh6*, and *Pln*) (f, g, i, j) or the fibroblast gene *Col3a1* (h) on mock and MGT‐transduced MEFs and AEFs on D11 expressed as % to Input and comparisons to corresponding mock for each gene. Each point on the plot indicates individual measurements and mean ± s.d of *n* = 3–6 biological replicates from three independent or one representative experiment. *p* values were calculated by unpaired t test or one‐way ANOVA followed by post‐hoc Tukey's test. *p < 0.05; **p < 0.01. Graphs were created using GraphPad Prism (RRID: SCR_002798) and Perseus (RRID: SCR_015753) (a–e).

To obtain insights into the age‐related remodeling of chromatin marks during DCC of fibroblasts, we performed mass spectrometry‐based bulk analysis of histone posttranslational modifications (PTMs, lysine acetylation, and methylation marks) in transdifferentiated MEFs and AEFs, along with the HL‐1 mouse cardiomyocyte cell line (Figure 2a and Table S2). PCA showed a clear separation in component 1 between fibroblasts (regardless of age and transduction) and the HL‐1 cardiomyocytes and a less pronounced separation between transdifferentiated MEFs (MGT) and the remaining fibroblasts in component 2 (Figure S1g), indicative of chromatin remodeling in transdifferentiated MEFs. Hierarchical clustering of differentially modified histone PTMs revealed two groups, that is, a first cluster (21/42 total modifications) mainly composed of histone 3 (H3) methylated residues and H4K20me2, with increased levels in fibroblasts; and a second cluster (21/42 total modifications) with increased levels in HL‐1 cells, which contains several histone H3 and H4 acetylated peptides (Figure 2a), highlighting that compared to fibroblasts, HL‐1 chromatin is enriched in acetylated and deprived in methylated H3/H4 residues (Figure 2a,b). We observed histone PTMs compatible with transcriptional silencing at D11, especially in MGT‐transduced MEFs, with an increased abundance of H3K27me3K36me1 (Figure S1h) and decreased abundance of H3K4me3 (Figure S1i, significant difference between AEFs MGT and MEFs MGT), which are typically associated with active promoters. Significant changes in histone PTMs were observed, particularly in MGT‐transduced MEFs (MEFs MGT/mock) and between MGT‐transduced embryonic and adult fibroblasts (AEFs MGT/MEFs MGT), with an increase in H3K9me1 levels (Figure 2c) and a decrease in H3K27me1 levels (Figure S1j) in MGT‐transduced embryonic fibroblasts. Differences in the chromatin of adult and embryonic parental fibroblasts were also detected, with MEFs presenting lower H3K27me2 levels (Figure 2d). Interestingly, a pronounced decrease in H4K20me2 levels, resembling those in HL‐1 cells, was detected in both embryonic and adult transdifferentiated fibroblasts (Figure 2e).

Next, we determined the physical occupancy of histone modifications at specific promoters of cardiac and fibroblast genes with relevance to DCC using chromatin immunoprecipitation (ChIP)‐qPCR in MGT‐transduced MEFs and AEFs on D11. The cardiac gene *Tnnt2* exhibited a significant decrease in the repression mark H3K27me3 (Figure 2f) and increase in the activation mark H3K4me3 (Figure 2g) in MGT‐transduced MEFs (compared to mock), whereas for MGT‐transduced AEFs, a significant increase in the repression mark H3K27me3 (Figure 2f) and no changes in H3K4me3 (Figure 2g) were observed, in agreement with the transcript levels of *Tnnt2* in MGT‐transduced MEFs and AEFs (Figure 1d). Similarly, ChIP‐qPCR revealed re‐patterning of the fibroblast marker *Col3a1*, demonstrating decreased H3K4me3 levels in MGT‐transduced MEFs (Figure 2h), paralleled by a reduction in mRNA expression at D11 (Figure 1e), with no major changes in MGT‐transduced AEFs. No differences were observed in H3K27me3 levels at *Col3a1* in the MGT‐transduced MEFs or AEFs (Figure S1k). Regarding the histone PTMs H3K9me1 and H4K20me2 with major alterations in the chromatin of transdifferentiated MEFs (Figure 2c,e), no significant differences in fibroblast (*Col3a1* and *Postn*) (Figure S1l,m) or cardiac (*Tnnt2*, *Myh6*, or *Pln*) (Figure 2i,j) genes were observed for MGT‐transduced MEFs, despite the lower abundance of H4K20me2 in *Tnnt2* compared to that in MGT‐transduced AEFs (Figure 2j). However, MGT‐transduced adult fibroblasts showed a higher abundance of both H3K9me1 and H4K20me2 marks at cardiac promoters (*Tnnt2*, *Pln*, *or Myh6*) (Figure 2i,j), which, together with the reduced mRNA expression level (Figure 1d and Figure S1c), suggests a putative role for H3K9me1 and H4K20me2 as repressive marks at cardiac loci. H3K9me1 and H4K20me2 showed increased enrichment at the promoter of *Postn* in MGT‐transduced AEFs (Figure S1l,m), which was not parallel to the changes in mRNA expression (Figure 1e). No differences in the expression of enzymes responsible for the deposition of H3K9me1 or H4K20me2 marks (*Setdb1* and *Kmt5b*, respectively) were observed between the conditions in the RNA‐seq data (Figure S1n). These results show significant epigenetic alterations in DCC with age and highlight H4K20me2 and H3K9me1 as putative repressive marks at cardiac loci that are relevant for DCC.

### 2.3 Transdifferentiation of mouse fibroblasts into iCMs is accompanied by metabolic and bioenergetic transitions resembling those present in cardiomyocytes

**FIGURE 3:**
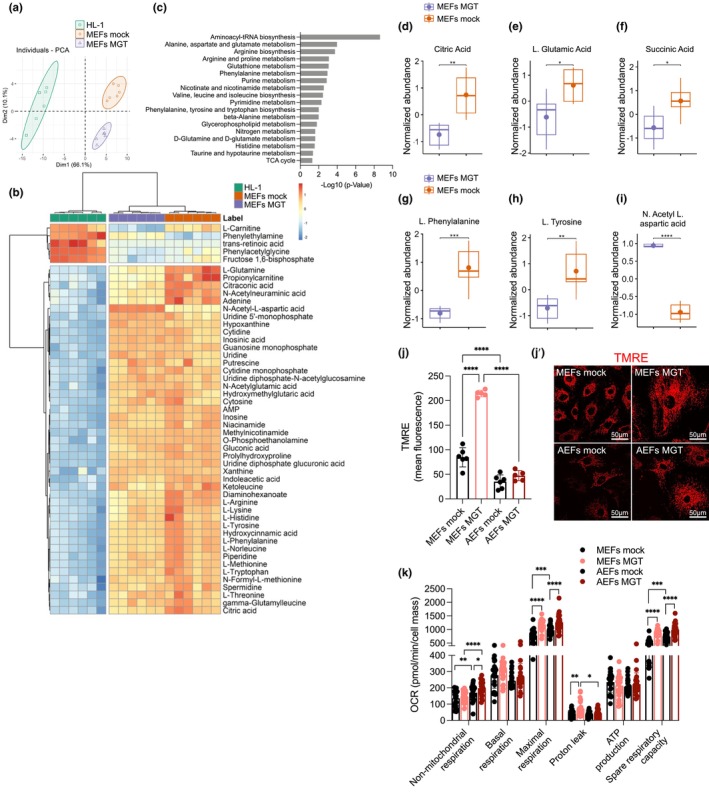
Transdifferentiation of mouse fibroblasts into iCMs is accompanied by metabolic and bioenergetic transitions resembling those present in cardiomyocytes. (a–i) LC–MS‐based untargeted metabolomics bulk analysis of MEFs mock or MGT‐transduced on D11, and the HL‐1 mouse cardiomyocyte cell line. PCA analysis score plot of the metabolite for the indicated groups (a). Two‐dimensional hierarchical clustering heatmap of the of the top 50 metabolites (*p* value <0.05) relative abundance, with the samples (on top) and identified species (left) (b). Top 18 significantly altered metabolic pathways constituted by the most differently expressed metabolites (*p* value <0.05), expressed in terms of −log10 of MEFs mock and MGT transduced (c). Selected box‐plot analysis relative abundances of citric acid (d), L‐glutamic acid (e), succinic acid (f), L‐phenylalanine (g), L‐tyrosine (h) and N‐acetyl‐L‐aspartic acid (i) comparison between MEFs mock (orange boxplots) and MEFs MGT (purple boxplots). (j‐j') TMRE live cell imaging and quantification depicted as relative mean fluorescence intensity (j) and representative images (j') in MEFs and AEFs mock or MGT transduced at D12. (k) Cellular oxygen consumption rate (OCR) (non‐mitochondrial‐, basal‐, maximal‐, proton leak‐ and ATP‐linked OCR, and spare respiratory capacity) in MEFs and AEFs mock or MGT transduced at D11. Data was obtained using Seahorse XF96 Cell Mito Stress Test and normalized to cell mass using the sulforhodamine B (SRB) assay. Each point on the plot indicates individual measurements and mean ± s.d of *n* = 3–6 biological replicates from one representative experiment (a–j) or *n* = 26–28 technical replicates from three independent experiments (k). *p* values were calculated by one‐way ANOVA and Kruskal‐Wallis tests with False Discovery Rate multiple comparisons test by post‐hoc Tukey's or Dunn's test. *p < 0.05; **p < 0.01; ***p < 0.001; ****p < 0.0001. Graphs were created using GraphPad Prism (RRID:SCR_002798), R package pheatmap (RRID:SCR_016418) and R package ggplot2 (RRID:SCR_014601) (a, b, d–i). Scale bars, 50 μm in (j').

Next, we performed LC–MS‐based metabolomics to evaluate metabolic alterations during the DCC of embryonic fibroblasts (Table S3). PCA (Figure 3a) showed clustering of samples into three groups (HL‐1, MEFs mock, and MGT), with a clear separation of MEFs mock and MGT along Dim2, revealing that transdifferentiation is accompanied by alterations in the metabolome. Nevertheless, the separation at Dim1 was indicative of a clear distinction between mouse cardiomyocytes (HL‐1) and MEFs, regardless of MGT transduction. Two‐dimensional hierarchical clustering from univariate analysis of the 50 most significantly modulated metabolites (Figure 3b) revealed a primary split in the upper hierarchical dendrogram, with HL‐1 cells clustered separately from MEFs (denoted in the PCA). The samples clustered into two groups based on relative abundance, that is, a smaller cluster of only five metabolites with higher abundance in HL‐1 cells and a larger group containing the remaining metabolites whose levels were increased in MEFs (Figure 3b). In both clusters, MEFs mock and MGT exhibited significant differences, where, in some cases, MGT‐transduced MEFs resembled HL‐1 cells, with increased levels of phenylethylamine (Figure S2a) and decreased levels of L‐glutamine (Figure S2b) and adenine (Figure S2c). Differential metabolic pathway analysis identified alanine, aspartate, and glutamate (*p* value <0.0001), phenylalanine (*p* value <0.001), nicotinate and nicotinamide (*p* value <0.01), phenylalanine, tyrosine, and tryptophan biosynthesis (*p* value <0.01), and taurine and hypotaurine metabolism (*p* value <0.05), related to the TCA cycle and anaplerotic reactions, including glutaminolysis, as the most significantly altered pathways (Figure 3c, Figure S2d, and Table S4). Of note, compared to the mock, MGT‐transduced MEFs exhibited a decrease in the relative abundance of the most significantly altered metabolites, including citric, L‐glutamic, succinic acid, L‐phenylalanine, and L‐tyrosine (Figure 3d–h), and an increased abundance of N‐acetyl‐L‐aspartic acid (Figure 3i), a metabolite synthesized in the mitochondria from the amino acid aspartate and acetyl‐CoA.

The link between mitochondrial respiration and cell cycle arrest has been established in postnatal cardiomyocytes (Lopaschuk et al., 1992). In agreement with this, cell cycle analysis by propidium iodide (PI) showed that MEFs MGT exhibited an increase in G2/M arrest and a decrease in G0/G1 and S phases (Figure S2e), despite no significant suppression of mitotic cell cycle genes by RNA‐seq (Table S1). Interestingly, AEFs MGT showed minor changes in the cell cycle and expected arrest as compared to MEFs (decreased proportion in G0/G1, S and increased proportion in G2/M, Figure S2e). Analysis of live TMRE staining, indicative of polarized mitochondria, revealed an increased intensity in MGT‐transduced MEFs compared to that in mock or AEFs MGT (Figure 3j,j'). To gain further insights into the bioenergetic switch during DCC, we measured the mitochondrial oxygen consumption rate (OCR) using Seahorse XF analysis (Figure 3k, Figure S2f). Both embryonic‐ and adult‐MGT‐transduced fibroblasts showed increased maximal mitochondrial respiration and spare respiratory capacity (Figure 3k), resembling the higher respiratory profile of HL‐1 cells (Figure S2f). Transdifferentiation of embryonic fibroblasts into iCMs was accompanied by a unique increase in proton leakage (Figure 3k), whereas higher non‐mitochondrial respiration was observed in MGT‐transduced adult fibroblasts (Figure 3k). No major differences in ECAR, an indirect indication of glycolytic flux (Figure S2g), or the overall energetic profile (OCR/ECAR, Figure S2h) were observed. Overall, these data suggest that, in parallel to cell cycle arrest, decreased engagement in anabolic pathways and increased reliance on the TCA cycle to fuel mitochondrial respiration occur during the MGT‐induced transdifferentiation of fibroblasts into iCMs.

### 2.4 Extensive remodeling of the mitochondrial network and mitophagy takes place during direct cardiac conversion

**FIGURE 4:**
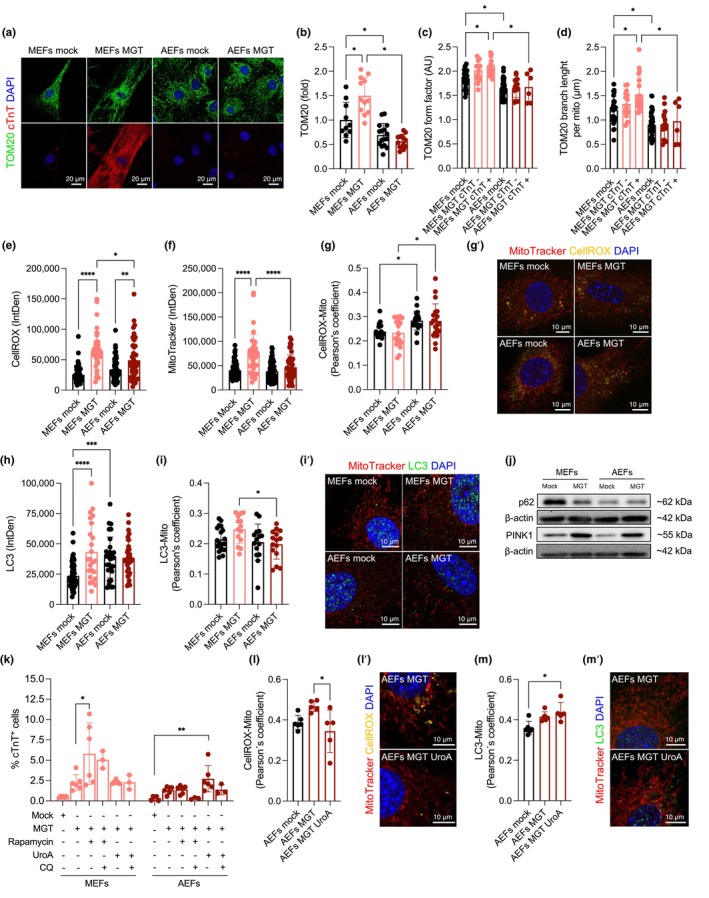
Extensive remodeling of the mitochondrial network and mitophagy takes place during direct cardiac conversion. (a–d) Representative images of mitochondria TOM20 and cardiac troponin (cTNT) (a), nuclei are labeled with DAPI (blue) and analysis of total TOM20 fluorescence corrected by cell area in MEFs and AEFs mock or MGT‐transduced (b). TOM20 form factor (shape, c) and branch length per mitochondria (d) in MGT‐transduced cTNT positive and negative cells at D11 (20 cTNT^+^/36 total cells for MEFs MGT and 6 cTNT^+^/22 total cells for AEFs MGT) or mock cells. (e–g') Quantification of CellROX (ROS) (e) and MitoTracker red (f) per cell (integrated density, IntDen), colocalization of CellROX in the mitochondria (Pearson's coefficient) (g) and representative images of MitoTracker red and CellROX, nuclei are labeled with DAPI (blue) (g') in MEFs and AEFs mock or MGT transduced at D11. (h–i') Analysis of LC3 signal per cell (IntDen) (h), colocalization of LC3 in the mitochondria (Pearson's coefficient) (i) and representative images of MitoTracker red and LC3, nuclei are labeled with DAPI (blue) (i') in MEFs and AEFs mock or MGT transduced at D11. (j) Immunoblotting analysis for p62, PINK1 and β‐Actin in whole cell extracts from MEFs and AEFs mock or MGT transduced at D11 (uncropped blots in Figure S6). (k–m') Flow cytometry quantification of the percentage of cardiac troponin (cTnT) positive cells (k), quantification of CellROX (l) and LC3 (m) colocalization in the mitochondria (Pearson's coefficient) and representative images (l', m') in MEFs and AEFs mock or MGT transduced at D11 untreated or supplemented with rapamycin (10 nM) or urolithin A (UroA, 5 μM) or chloroquine (CQ) (25 μM, added for 4 h before cell harvest) in the indicated conditions. Each point on the plot indicates individual measurements and mean ± s.d of *n* = 6–43 (b–d), *n* = 20–83 (e–i), *n* = 5–6 (l, m) cells analyzed or *n* = 3–6 biological replicates (j, k) from one representative or two independent experiments. *p* values were calculated by Kruskal Wallis with false discovery rate correction and one‐way ANOVA (b‐d) and Tukey's post‐test for multiple comparisons.*p < 0.05; **p < 0.01; ***p < 0.001; ****p < 0.0001. Graphs were created using GraphPad Prism (RRID:SCR_002798). Scale bars, 20 μm (a) and 10 μm in (g', i', l', m').

The extent of mitochondrial remodeling during the transdifferentiation of fibroblasts into iCMs remains largely unknown. Immunofluorescence analysis of TOM20‐labeled mitochondria revealed increased total fluorescence in MGT‐transduced MEFs (Figure 4a,b), with no significant alterations in MGT‐transduced AEFs. No major differences in the number of TOM20 particles were observed (Figure S3a), whereas both MGT‐transduced MEFs and AEFs present an increased TOM20 total area (Figure S3b), indicative of size, resembling the conditions present in HL‐1 cells. Immunofluorescence revealed that MGT‐transduced MEFs exhibited increased expression of the sarcomeric protein cTNT (compared to mock or MGT‐transduced AEFs) (Figure 4a and Figure S3c), in agreement with the flow cytometry results (Figure 1b,b'), despite the lack of well‐defined structures at this stage (D11). MGT‐induced transdifferentiation of MEFs was accompanied by significant remodeling of the mitochondrial network, particularly when analyzing cTNT+ cells (iCMs), with a significant increase in the form factor, indicative of an elongated shape (Figure 4c), and several parameters of connectivity, such as branch length (Figure 4d) and junctions per mitochondrion (Figure S3d), with minor differences in MGT‐transduced AEFs expressing cTNT.

During CM maturation, the reliance on mitochondrial respiration is accompanied by the production of reactive oxygen species (ROS) and the adoption of FAs and pyruvate as the main energetic substrates (Lehman & Kelly, 2002). AEFs MGT exhibited more lipid droplets than MEFs MGT (Figure S3e), which may suggest differential usage and mobilization of lipids by adult transdifferentiated cells. Analysis of CellROX, indicative of cellular ROS (Figure 4e), revealed increased levels during transdifferentiation of both embryonic and adult fibroblasts, with MEFs MGT presenting the highest level, in agreement with proton leak measurements (Figure 3k) and higher MitoTracker staining, indicative of mitochondrial mass (Figure 4f), in line with previous observations (Figures 3j and 4a–d). Strikingly, adult fibroblasts, both mock‐ and MGT‐transduced, showed increased colocalization of ROS in the mitochondria (Figure 4g,g'). Mitochondrial network remodeling depends on processes such as mitochondrial biogenesis, mitophagy, and mitochondrial dynamics. No differences in the expression of peroxisome proliferator‐activated receptor gamma coactivator 1‐alpha (PGC‐1α), the master regulator of mitochondrial biogenesis, were observed upon transdifferentiation (Figure S3f,f'). Analysis of the autophagy marker LC3 revealed higher levels in adults compared to embryonic fibroblasts and increased levels in transdifferentiated MEFs (Figure 4h). Adult transdifferentiated fibroblasts exhibited fewer LC3 puncta in the mitochondria (compared to MGT‐transduced MEFs) (Figure 4i,i'), indicative of defective mitophagy. Consistent with this, the transdifferentiation of MEFs was accompanied by a decrease in the expression of p62 and an increase in the expression of PINK1, a ubiquitin ligase that initiates mitophagy (Figure 4j and Figure S3g), whereas less pronounced effects were observed in AEFs MGT. Next, we sought to determine whether mitophagy activation facilitates DCC in adult fibroblasts. Treatment with rapamycin (10 nM), which was previously shown to promote autophagy and DCC in neonatal mouse cardiac fibroblasts (CFs) (Wang et al., 2020), increased the percentage of cTnT+ cells in MEFs MGT (Figure 4k, Figure S3h), with no major impact on AEFs MGT. Conversely, supplementation of the mitophagy inducer urolithin A (UroA, 5 μM) increased the percentage of cTnT+ cells in AEFs MGT to the level of untreated MEFs MGT (Figure 4k and Figure S3h, mean 2.72% versus 2.26%, respectively), and incubation with the lysosomal inhibitor chloroquine (CQ) (25 μM) blunted this effect. Importantly, AEFs MGT supplemented with UroA showed decreased CellROX (Figure 4l,l') and increased LC3 (Figure 4m,m') puncta in the mitochondria. Overall, these results suggest that the activation of mitophagy and subsequent clearance of mitochondria under oxidative stress may be required for the efficient transdifferentiation of adult fibroblasts into iCMs.

### 2.5 Metabolic modulation can bypass epigenetic and age‐associated barriers during DCC

**FIGURE 5:**
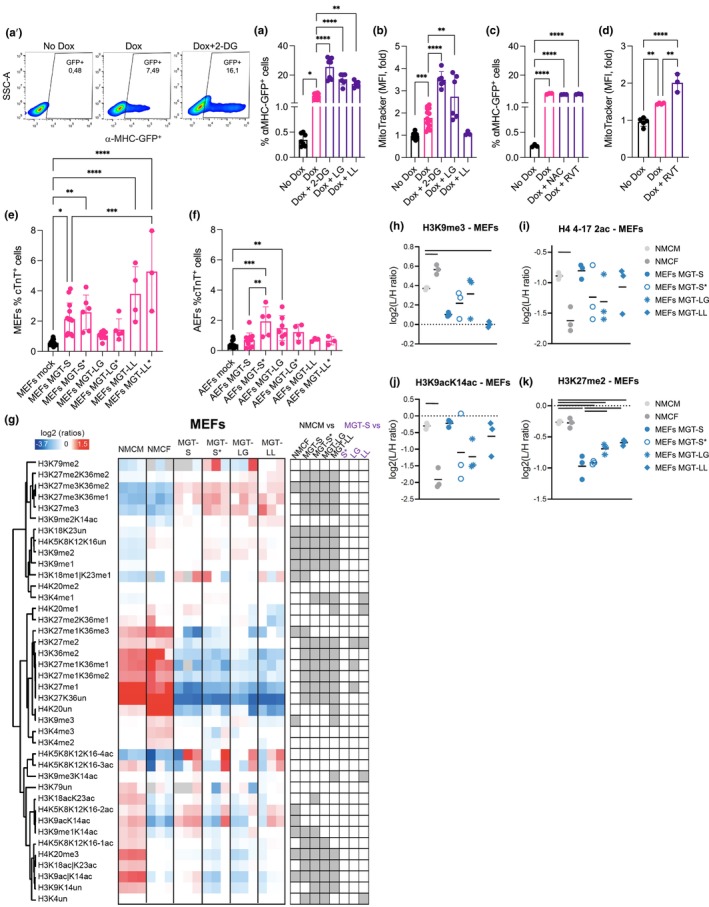
Metabolic modulation can bypass epigenetic and age‐associated barriers during DCC. (a–a') Flow cytometry representative plots (a') and quantification of the percentage of αMHC‐positive cells (a) in icMEFs with no Dox or Dox for 3 days in the presence of 2‐Deoxy‐D‐glucose (2‐DG, 1 mM), low glucose (LG: Glucose 1 mM, FBS 10%) or low lipids (LL: Glucose 25 mM, FBS 1%) growth medium. (b) Flow cytometry quantification of mitochondrial mass by MitoTracker deep red staining depicted as relative median fluorescence intensity (MFI) in icMEFs with no Dox or Dox for 3 days in the presence of 2‐DG, LG or LL growth medium. (c, d) Flow cytometry quantification of the percentage of αMHC‐positive cells (c) and mitochondrial mass by MitoTracker deep red staining depicted as the fold change of the relative MFI (d) in icMEFs with no Dox or Dox for 3 days in the presence of N‐acetylcysteine (NAC, 5 mM) or resveratrol (RVT, 20 nM). (e, f) Flow cytometry quantification of the percentage of cardiac troponin (cTnT) positive cells in MEFs (e) and AEFs (f) mock or MGT transduced in the presence of standard medium (S: Glucose 25 mM, FBS 10%), low glucose (LG: Glucose 1 mM, FBS 10%) or low lipids (LL: Glucose 25 mM, FBS 1%) only or supplemented with sodium acetate and α‐KG (S*, LG*, LL*, sodium acetate 5 mM and α‐KG 1.5 mM) assayed on D12. (g–k) Mass spectrometry‐based histone acetylation and methylation proteomic bulk analysis in neonatal mouse cardiac myocytes (NMCM) and fibroblasts (NMCF) and MGT‐transduced MEFs on D11 in the presence of standard medium (S) only or supplemented with sodium acetate and α‐KG (S*), low glucose (LG) or low lipids (LL) growth medium. Heatmap display of histone PTM levels clustered based on Pearson's correlation, grey squares indicates peptides that were not quantified. The panel on the right shows significant changes (grey) between NMCM and MGT‐S and the indicated comparisons (g). Display of selected histone modified peptides showing levels of tri‐methylated form of H3 lysine 9 (h), bi‐acetylated form of H4 lysine 4–17 (i), mono‐acetylated H3 lysine 9 and 14 (j) and di‐methylated form of H3 lysine 27 (k) in the indicated groups, the bar indicates a *p* value <0.05. Each point on the plot indicates individual measurements and mean ± s.d of *n* = 3–12 biological replicates from two independent or one representative experiment. *p* values were calculated by one‐way ANOVA followed by post‐hoc Tukey's test. *p < 0.05; **p < 0.01; ***p < 0.001; ****p < 0.0001 in respect to Dox only (a, b) or between the indicated groups. Graphs were created using GraphPad Prism (RRID:SCR_002798), Perseus (RRID:SCR_015753) (g–k) and flow cytometry plots (a') with FlowJo (RRID:SCR_008520).

To test the impact of mitochondria and metabolic modulation in DCC, we took advantage of a transformed cell line derived from MEFs and harboring an α‐myosin heavy chain (MHC)‐eGFP reporter under the control of doxycycline (Dox) (Vaseghi et al., 2016), hereafter referred to as icMEFs. In the presence of Dox, the expression of MGT factors induced cardiac fate, which was readily observed by GFP expression using flow cytometry and fluorescence microscopy (Figure 5a',a and Figure S4a). Importantly, we observed similar mitochondrial remodeling as in transdifferentiated primary fibroblasts, including an increased total mitochondrial area, form factor, and branches per mitochondrion (Figure S4b–d). Supplementation of Dox, together with the glycolysis inhibitor 2‐deoxy‐D‐glucose (2‐DG, 1 mM), low glucose (LG: glucose 1 mM, FBS 10%), or low lipids (LL: glucose 25 mM, FBS 1%) growth medium increased the percentage of αMHC‐eGFP+ cells (Figure 5a,a'). The addition of Dox promoted an increase in mitochondrial mass in icMEFs, as assessed by MitoTracker staining (Figure 5b), similar to that observed in MGT‐transduced primary MEFs (Figure 4f), which was further enhanced by most metabolic manipulations, except LL (Figure 5b). Moreover, icMEFs have higher ROS levels (Figure S4e), as previously observed for MEFs MGT (Figure 4e); however, treatment with the cellular antioxidant N‐acetylcysteine (NAC, 5 mM) had no impact on the percentage of αMHC‐eGFP+ cells (Figure 5c). Further, despite the increase in mitochondrial mass produced by supplementation with resveratrol (RVT, 20 nM) (Figure 5d), a known activator of PGC‐1α, no significant changes in the percentage of αMHC‐eGFP+ cells were observed (Figure 5c). These data suggest that forcing mitochondrial energy metabolism enhances DCC through mechanisms that do not necessarily rely on increased mitochondrial mass. In primary fibroblasts, supplementation with sodium acetate (precursor of acetyl‐CoA) and α‐KG (* sodium acetate 5 mM and α‐KG 1.5 mM) under low lipid (LL*) or standard (S*: glucose 25 mM, FBS 10%) conditions led to increase percentage of cTnT+ cells in MGT‐transduced MEFs and AEFs, respectively (Figure 5e,f), whereas LG medium had no impact.

To investigate whether metabolic manipulations enhance DCC by promoting epigenetic alterations, we performed histone PTMs bulk analysis using mass spectrometry in transdifferentiated MEFs and AEFs in different growth media and compared them with neonatal mouse cardiomyocytes (NMCMs) and cardiac fibroblasts (NMCFs) (Table S5). PCA clustered the samples into three groups, that is, NMCFs, NMCMs, and the remaining MEFs (Figure S4f) and AEFs (Figure S4g). A clear distinction was observed between MGT‐transduced MEFs or MGT‐transduced AEFs and neonatal mouse cardiac cells (both cardiomyocytes and fibroblasts) in component 1 (Figure S4f,g), and a less pronounced separation between mouse fibroblasts (regardless of origin, age, and growth medium supplementation) and NMCMs was observed in component 2 (Figure S4f,g). Despite the increased percentage of cTNT+ cells in MGT‐transduced MEFs in low lipids (MGT‐LL) alone or supplemented with sodium acetate and α‐KG (MGT‐S*, LL*) (Figure 5e), no major differences in the histone PTMs were observed when compared to the standard (MGT‐S) (Figure 5g). Nevertheless, the levels of certain histone marks in MGT‐transduced MEFs under standard (MGT‐S) or MGT‐LL conditions more closely resembled those of NMCMs and not those of NMCFs, such as decreased H3K9me3 levels (Figure 5h) and higher H4 lysine 4–17 2 ac (Figure 5i), and H3K9acK14ac levels (Figure 5j). Notably, MGT‐LL showed higher levels of H3K27me2 (Figure 5k) and H3K4un (unmodified) (Figure S4h) than MGT‐S and resembled NMCMs. For transdifferentiated AEFs, similar results were observed, as the increased percentage of cTNT+ cells achieved with MGT transdifferentiation supplemented with sodium acetate and α‐KG (MGT‐S*) (Figure 5f) was not accompanied by major differences in histone PTMs (Figure S4i). However, the levels of H3K9me1 (Figure S4j), H4 lysine 4–17 2 ac (Figure S4k), and H3K9acK14ac (Figure S4l) in MGT‐transduced AEFs under standard (MGT‐S) or sodium acetate and α‐KG supplementation (MGT‐S*) more closely resembled those of NMCMs and not NMCFs. Overall, these results indicate that metabolic modulation can improve the DCC of embryonic and adult fibroblasts and is accompanied by the remodeling of chromatin marks.

### 2.6 Dietary lipids improve DCC of adult fibroblasts ex vivo

**FIGURE 6:**
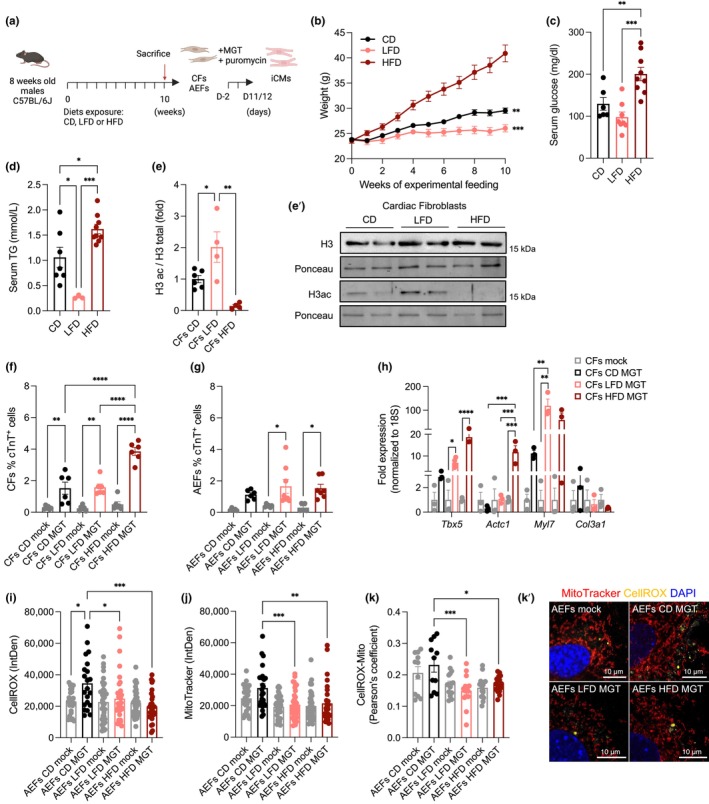
Dietary lipids improve DCC of adult fibroblasts ex vivo. (a) Schematic representation of the procedure. Adult male mice were subjected to high fat (HFD, 60 kcal% fat), low fat/high fructose (LFD, 5 kcal% fat and 65% kcal fructose) and control purified ingredient (CD, 10 kcal% fat) diets for 10 weeks. After sacrifice, cardiac and adult skin ear fibroblasts (CFs and AEFs, respectively) were isolated, seeded (D‐2) and ex vivo MGT transduced followed by puromycin selection, and analysed at D11/12. (b) Body weight (grams, g) of animals under control (CD), high fat (HFD) and low fat/high fructose (LFD) diets from week 0 to 10. (c, d) Serum glucose (c) and triglycerides (TG, d) concentration from CD, HFD and LFD animals at the end of the dietary regimen. (e–e') Immunoblotting analysis of total histone H3 and H3 pan‐acetylated displaying densitometric quantification of H3ac/H3 total (e) and representative blots (e', uncropped blots are shown in Figure S6) from histone‐enriched extracts of CFs isolated from animals under CD, HFD and LFD. (f, g) Flow cytometry quantification of the percentage of cardiac troponin (cTnT) positive cells in CFs (f) and AEFs (g) mock or MGT transduced from CD, HFD and LFD at D11. (h) qPCR analysis of the relative expression of the indicated genes in CFs isolated from animals under CD, HFD and LFD subjected to mock or MGT transduction at D11 (fold to corresponding mock). (i–k') Quantification of CellROX (ROS) (i), MitoTracker red (j) per cell (integrated density, IntDen), and mitochondria‐specific ROS puncta (Pearson's coefficient) (k) and representative images (k'), nuclei are labeled with DAPI (blue), in AEFs isolated from animals under CD, HFD and LFD and subjected to mock or MGT transduction at D11. Each point on the plot indicates individual measurements and mean ± s.d of *n* = 3–9 biological replicates (b–h) or *n* = 11–40 cells analyzed (i‐k) from one representative or two independent experiments. *p* values were calculated by one‐way ANOVA with False Discovery Rate followed by Tukey's post‐test for multiple comparisons. *p < 0.05; **p < 0.01; ***p < 0.001; ****p < 0.0001 with respect to HFD (b) or the indicated groups. Graphs were created using GraphPad Prism (RRID:SCR_002798) and schema (a) with BioRender.com. Scale bars, 10 μm in (k').

As dietary lipids can modulate histone acetylation levels in normal tissues (Carrer et al., 2017), we investigated their impact on DCC. We exposed young C57BL/6J adult male mice to an isocaloric high‐fat diet (HFD, 60% fat), low‐fat/high‐fructose diet (LFD, 5% fat and 65% fructose), or control diet (CD, 10% fat) for 10 weeks (Figure 6a). HFD‐animals showed a significant increase in body weight (compared to LFD‐ and CD‐fed animals) (Figure 6b) and increased serum glucose (Figure 6c) and triglyceride (TG) levels (Figure 6d); a pronounced decrease in TG levels was observed in LFD‐fed animals (Figure 6d).

We established ex vivo primary cultures of adult CFs and skin dermal ear‐derived fibroblasts (AEFs) of the animals at the end of the dietary regimen (Figure 6a). Adult CFs and AEFs derived from LFD‐fed and HFD‐fed animals presented a general decrease in the expression of key markers of activated fibroblasts such as *Postn* and *Fn1* (Figure S5a,b), whereas fibroblasts from HFD‐fed animals exhibited opposing levels of *Fap*, with decreased levels in CFs (Figure S5a) and increased expression in AEFs (Figure S5b).

Immunoblotting analysis revealed no differences in the levels of total histone H3 in CFs isolated from animals exposed to different diets (Figure S5c and Figure 6e'), whereas major alterations were observed in the levels of pan‐acetylated H3 (acetyl K9 + K14 + K18 + K23 + K27), with increased and decreased levels in CFs derived from LFD‐ and HFD‐fed animals, respectively (Figure 6e,e' and Figure S5d), suggesting that dietary lipids can result in long‐lasting histone PTMs.

MGT transduction of CD‐derived adult fibroblasts revealed a slight increase in the percentage of cTnT+ cells in CFs compared to that in AEFs (Figure 6f,g; mean 1.52% vs. 1.12%, respectively). Transdifferentiation of CFs from HFD‐fed animals produced a higher percentage of cTnT+ cells than mock‐infected or CD MGT (Figure 6f; mean 3.86%). For AEFs, an increased percentage of cTnT+ cells was observed in both MGT‐transduced cells derived from LFD‐ and HFD‐fed animals (compared to mock cells), in contrast to the lower efficiency of transdifferentiated CD AEFs (Figure 6g; mean 1.65% and 1.37%, respectively). Accordingly, qPCR analysis revealed increased expression of the cardiogenic reprogramming factor *Tbx5* and the cardiac genes *Actc1* and *Myl7* and a tendency for lower expression of the fibroblast marker *Col3a1* in MGT‐transduced CFs from LFD and HFD animals (Figure 6h). Similar results were observed for MGT‐transduced AEFs from HFD and LFD animals, with increased expression of *Tnnt2* and decreased expression of the fibroblast markers (*Col1a1* and *Eln*) (Figure S5e). Improvements in transdifferentiation induced by dietary lipids may be related to alterations in mitochondrial metabolism. No major differences in TMRE live staining were observed for MGT‐transduced AEFs derived from animals subjected to different dietary regimens (Figure S5f). Immunofluorescence analysis revealed higher ROS levels in transdifferentiated AEFs from CD and lower levels in MGT‐transduced AEFs from LFD and HFD (Figure 6i), which also showed decreased MitoTracker staining compared with CD MGT (Figure 6j). Strikingly, MGT‐transduced AEFs from the LFD and HFD groups showed reduced oxidative stress in the mitochondria (Figure 6k,k'), which may facilitate the transdifferentiation of adult fibroblasts.

## 3 DISCUSSION

Aging is a barrier to cell reprogramming, possibly because of abnormal chromatin states or the abundance and function of histone‐modifying enzymes (Pal & Tyler, 2016). Here, using MEFs and AEFs (from animals of different ages), we provide evidence that the direct reprogramming of fibroblasts into iCMs decreases with age. MGT‐transduced MEFs exhibit characteristics of reprogramming at higher levels than adult skin fibroblasts (i.e., increased % of cTNT+ cells, chromatin re‐patterning, and higher global transcriptional induction of cardiomyocytes and repression of fibroblast markers, expression of sarcomeric proteins, and spontaneous Ca^2+^ oscillations), despite the lack of fully reprogrammed mature iCMs at the time points analyzed, compatible with the early stages of cardiac reprogramming (Chen et al., 2012; Ieda et al., 2010; Kurotsu et al., 2020; Song et al., 2012; Wang et al., 2015; Zhou et al., 2017). DCC has been applied to several types of fibroblasts including adult tail tip (TTFs), MEFs, neonatal, and adult CFs. The use of adult dermal fibroblasts, despite presenting a lower reprogramming efficiency than that of CFs (Ieda et al., 2010; Wang et al., 2015; Song et al., 2012; Addis et al., 2013), can be translated into the future application of patient‐derived skin fibroblasts to generate iCMs for cell‐based heart therapy, avoiding gene delivery in situ and the risk of tumor formation from the alternative iPSC‐derived CMs strategy (Kojima & Ieda, 2017). Apart from the starting fibroblast population, several conditions influence DCC efficiency, as the reprogramming factors used and its stoichiometry (e.g., MGT or additional factors as Hand2, Nkx2.5, or Myocd), quality and type of virus encoding (retroviral, lentiviral, Sendai viral vectors), defined culture condition (e.g., iCM medium here or more complex formulations that accelerate iCM maturation), day of analysis, and cardiomyocyte markers used (i.e., cTNT, α‐MHC‐GFP reporter), making it difficult to compare among different studies. Nevertheless, the % of cTNT+ cells obtained upon MGT transduction of MEFs (mean 2.26%–2.62%) was comparable to that reported in previous studies using similar conditions (GMT, 1 week post‐transduction) (Christoforou et al., 2013; Muraoka et al., 2014). Although no previous studies have reported the use of adult skin dermal ear‐derived fibroblasts, MGT transduction of adult TTFs or CFs yielded a similar induction of cTNT+ cells as reported here (1.4%–2.9% cTNT+ α‐MHC‐GFP+ double positive cells for GMT or MGT transduction at 7–11 days) (Song et al., 2012; Wang et al., 2015). Importantly, supplementation with the mitophagy inducer UroA or exposure to dietary lipids increased the percentage of cTNT‐expressing cells in MGT‐transduced adult fibroblasts to the levels achieved in embryonic fibroblasts (AEFs 2.72% and CFs 3.86%). In an injured heart, early post‐myocardial infarction, resident CFs are activated and differentiate into myofibroblasts, leading to dynamic ECM changes and maladaptive tissue remodeling (Gibb et al., 2020). We assessed the profile of the parental cells used for reprogramming. Compared to embryonic fibroblasts, adult fibroblasts (CFs and AEFs from 5 to 12 month old animals) present decreased expression of activated or myofibroblast markers, suggesting that the origin (tissue) and age of the starting fibroblast population critically impact DCC.

During reprogramming, early rapid activation of the cardiac program (D3) and later progressive suppression of fibroblast fate (D10) at both the epigenetic and transcriptional levels have been reported in transdifferentiated cardiac and tail tip fibroblasts (Ieda et al., 2010; Liu et al., 2016; Zhou et al., 2017). In agreement with this, our results provide evidence for the re‐patterning of H3K4me3 and H3K27me3 marks at the cardiac *Tnnt2* and fibroblast *Col3a1* promoters in MGT‐transduced embryonic fibroblasts, which is paralleled by alterations in the expression of *Tnnt2* and *Col3a1*. Histone PTMs analysis highlighted substantial chromatin modifications in MGT‐transduced fibroblasts with increased levels of H3K9me1, H3K27me3K36me1, and H3K27me1 and a remarkable decrease in H4K20me2 in both MGT‐transduced MEFs and AEFs, resembling HL‐1 cardiomyocytes, but could not provide insights into its abundance at specific promoters relevant to DCC. ChIP‐qPCR analysis revealed re‐patterning of H3K9me1 and H4K20me2 marks at promoters of cardiac genes, especially in adult transdifferentiated cells, that together with the mRNA expression level, suggest a putative repressive role. Contrasting outcomes have been attributed to the manipulation of H3K9me1 in DCC, either impairing or enhancing reprogramming at early and later time points, respectively (Hirai & Kikyo, 2014). H4K20me2 is enriched at sites of DNA damage, and lower levels of H4K20me2 are associated with decreased cell cycle progression (Paquin & Howlett, 2018). Further studies are required to clarify the roles of H3K9me1 and H4K20me2 in DCC.

Mitochondrial fatty acid utilization increases chromatin oxidative stress and contributes to cell cycle arrest and CMs maturation (Menendez‐Montes et al., 2016). MS‐based metabolomics revealed significant alterations in the TCA cycle and glutaminolysis, suggestive of increased fueling of mitochondrial respiration, which was further confirmed by a higher maximal OCR and spare respiratory capacity in MGT‐transduced fibroblasts, which may reflect increased oxidative capacity, substrate provision, or altered mitochondrial homeostasis during DCC.

We further showed that MGT‐transduced embryonic fibroblasts displayed branched and complex mitochondrial networks that also occur during CMs maturation (Lopaschuk et al., 1992). Remodeling of mitochondrial morphology and dynamics affects several mitochondrial functions, such as bioenergetics, apoptosis, and ROS production (Hong et al., 2022). We showed that transdifferentiated fibroblasts presented increased levels of ROS‐labeling dyes and proton leakage. ROS (such as superoxide and hydrogen peroxide) act as secondary messengers and play important roles in cell fate regulation and reprogramming (Maryanovich & Gross, 2013; Wang et al., 2021). However, exacerbated oxidative stress may be detrimental to cellular homeostasis. We observed that MGT‐transduced adult fibroblasts exhibited increased oxidative stress in the mitochondria, suggestive of accumulated damage (Hong et al., 2022), decreased levels of activated mitophagy markers (e.g., lower localization of LC3 in the mitochondria), and that supplementation with the mitophagy inducer UroA promoted DCC and improved mitophagy markers. Indeed, PINK1‐Parkin‐mediated mitophagy is essential for the fetal maturation of CMs (Gong et al., 2015).

Several mitochondria‐derived metabolites (e.g., TCA cycle intermediates) serve as cofactors or substrates for chromatin‐modifying enzymes, linking metabolism to epigenetic and transcriptional regulation (Intlekofer & Finley, 2019). We show that inhibition of glycolysis or deprivation of lipids alone or together with supplementation of sodium acetate (converted into acetyl‐CoA by ACSS2) and α‐KG consistently increased DCC in vitro in MGT‐transduced primary fibroblasts and icMEFs. Moreover, adult skin and cardiac fibroblasts derived from both HFD‐ and LFD‐fed animals exhibited increased DCC. Histone PTM analysis revealed similarities between MGT‐transduced embryonic fibroblasts generated under low‐lipid conditions and NMCMs; higher levels of H3K27me2 and H3K4un suggested that lipid deprivation may increase DCC via epigenetic remodeling. This is consistent with previous studies on pluripotent cells, where low exogenous lipid levels promoted de novo lipogenesis and the contribution of acetyl‐CoA to histone acetylation (Cornacchia et al., 2019). Future genome‐wide studies using ATAC‐seq or ChIP‐seq should provide additional insights into the impact of metabolic modulation on chromatin regions relevant to iCM reprogramming.

Pharmacological manipulations that promote histone acetylation and demethylation increase DCC efficiency in vitro (Singh et al., 2020; Wang et al., 2021). We observed that CFs isolated from HFD‐ and LFD‐fed animals present altered levels of histone H3 acetylation (decreased and increased, respectively), in accordance with the literature (Carrer et al., 2017). Both nutritional manipulations increased the percentage of cTNT+ cells upon transdifferentiation ex vivo, reinforcing that complex chromatin remodeling (as evidenced by our histone PTMs analysis, Figures 2 and 5) regulates DCC. Remarkably, similar to UroA supplementation, transdifferentiated LFD‐ and HFD‐derived fibroblasts exhibited less oxidative stress in the mitochondria, linking decreased mitochondrial damage to improved DCC in adult fibroblasts.

Most of our studies were performed on bulk MGT‐transduced fibroblasts (including RNA‐seq, MS‐based histone PTMs, and metabolomics), which may have contributed to less pronounced differences and lower iCMs maturation. Future employment of strategies for iCMs purification, such as molecular beacon selection coupled with FACS sorting (Bachamanda Somesh et al., 2023) and optimized procedures (maturation medium, later time‐points), may provide a translational opportunity for the use of primary skin fibroblasts, including those from patients with CVDs and dyslipidemia, for DCC and cell‐based heart therapies.

Overall, our study revealed pronounced metabolic and epigenetic remodeling in cell lineage conversion during transdifferentiation and suggested that the availability of mitochondria‐derived metabolites favors chromatin landscape transitions driving DCC, highlighting the dynamic crosstalk between mitochondria and epigenetics. Moreover, we showed that the activation of mitophagy and metabolic modulation that reduces oxidative damage in the mitochondria improves DCC in adult fibroblasts, which can be further exploited for heart regenerative strategies.

## 4 EXPERIMENTAL PROCEDURES

### 4.1 Mice studies

Male mice C57BL/6J were purchased from the Charles River Laboratories. All animal studies were performed at the iBiMED animal facility at the University of Aveiro, reviewed and approved by the Ethical Commission (CEDCM‐BEA), and licensed by the national regulatory agency DGAV. Mice were housed and handled in accordance with the standard protocols of the European Animal Welfare Legislation Directive 2010/63/EU (European Commission, 2016). Seven‐week‐old male C57BL/6J mice were acclimatized for 1 week prior to the initiation of the study. Mice were randomly assigned to dietary regimens, including high‐fat (HFD, 60% fat, D12492i), low‐fat/high‐fructose (LFD, 5% fat and 65% fructose, D22060205i), and control purified ingredient (CD, 10% fat, D12450Ki, all from Research Diets) diets for 10 weeks (*n* = 8–9 animals per diet), using weight‐matched littermate mice. Body weights were recorded weekly. The mice were sacrificed by cervical dislocation. At necropsy, blood was collected by cardiac puncture into centrifuge tubes, centrifuged for 15 min at 1600 ***g***, and the serum was stored at −80°C until analysis. The heart was dissected, and skin tissue samples from the ear were collected and kept in PBS 1× at 4°C before fibroblast isolation.

### 4.2 Determination of serum glucose and triglycerides

Serum glucose and triglyceride levels were measured using the Liquick Cor‐GLUCOSE 60 Kit (Cormay Diagnostics 2–201) and Liquick Cor‐TG 60 Kit (Cormay Diagnostics 2‐253) following the manufacturer's instructions. The absorbance was read at 500 and 550 nm using a Tecan Infinite M200 (Tecan) plate reader.

### 4.3 Culture of mammalian cell lines and mouse primary fibroblasts

Human Embryonic Kidney 293 T (HEK 293 T) were purchased from ATCC between 2012 and 2016, and mouse HL‐1 (Claycomb et al., 1998) and icMEFs (Vaseghi et al., 2016) were kindly provided by Dr. Christian Bär and Dr. Li Qian, respectively. All lines were tested as being mycoplasma‐free, and authenticated based on morphological examination and consistent in vitro performance. Cell lines and primary fibroblasts were cultured in DMEM (Gibco) high glucose (4.5 g/L glucose) or low glucose (1 g/L glucose), supplemented with 10% (v/v) heat‐inactivated FBS (Gibco), 1 mM sodium pyruvate, and 1% antibiotic‐antimycotic (Gibco) at 37°C and 5% CO_2_ atmosphere. HL‐1 cells were cultured in Claycomb medium (SIGMA) supplemented with 10% FBS, 1% penicillin–streptomycin (Gibco), 0.1 mM norepinephrine (SIGMA), and 2 mM L‐glutamine (Gibco). iCMs were cultured in DMEM/M199 (Zenbio) (4:1) with 10% FBS, 1 mM sodium pyruvate, and 1% antibiotic‐antimycotic from day 1 post‐transduction (D1) until D12, and then replaced with RPMI 1640 medium (Gibco) supplemented with B27 until D25 (Wang et al., 2015).

### 4.4 Direct cardiac conversion of fibroblasts into induced cardiomyocytes

Fibroblasts were retrovirally infected in accordance to the method proposed by Wang et al. (Wang et al., 2015). Briefly, on day 1.5 × 10^6^ HEK 293 T cells were seeded into a 10 cm plate. Four micrograms of plasmid DNA of either pMX‐puro‐MGT (Addgene, #111809), a polycistronic vector that expresses the reprogramming factors Mef2c, Gata4, and Tbx5 (MGT), or pBabe‐puro (an empty back bone vector, used as a mock; Addgene, #1784) was mixed with 4 μg pCL‐Ampho (retroviral packaging plasmid), together with 576 μL Opti‐MEM (Gibco) media containing 24 μL of X‐tremeGENE 9 DNA (Roche) and added to HEK cells. The medium was changed the next day (day 2), and fibroblasts were seeded at a density of 1.6 × 10^5^ cells per well in 6‐well plates or 1.2 × 10^6^ cells in 10 cm plates previously coated with 0.1% gelatin porcine (SIGMA). Virus containing‐medium was collected 48 h after transfection (day 3), followed by filtration through a 0.45 μm polyethersulfone filter and supplementation with polybrene (Millipore, 10 μg/mL), and added to target cells. This step was performed twice daily for 2 days. On day 5 (day 1 post‐transduction; D1), the virus‐containing medium was replaced with growth medium and changed every 3–4 days until analysis. For positive selection, puromycin (SIGMA, 2 μg/mL) was added to target cells on D1 for 3 days and maintained at a concentration of 1 μg/mL.

For growth medium manipulation, the medium was replaced on D4 by standard (S: glucose 25 mM, FBS 10%), low glucose (LG: glucose 1 mM, FBS 10%), or low lipid (LL: glucose 25 mM, FBS 1%) only, or supplemented with sodium acetate and α‐KG (sodium acetate 5 mM and α‐KG 1.5 mM) (S*, LG*, LL*). Rapamycin (Tocris Bioscience, 10 nM) or urolithin A (UroA, SIGMA, 5 μM) was added on D4 until analysis. Chloroquine (CQ, SIGMA 25 μM) was added 4 h before cell harvest. icMEFs were seeded at a density of 1.5 × 10^4^ cells per well in 24‐well plates previously coated with 0.01% gelatin porcine (SIGMA). The following day (day 0), growth medium was replaced with medium supplemented with doxycycline (Dox, SIGMA, 2 μg/mL) only or supplemented with 2‐deoxy‐d‐glucose (2‐DG, SIGMA, 1 mM), N‐acetylcysteine (NAC, SIGMA, 5 mM), resveratrol (RVT, SIGMA, 20 nM), or substituted by low glucose (LG: glucose 1 mM, FBS 10%) or low lipids (LL: glucose 25 mM, FBS 1%) for 3 days.

### 4.5 Flow cytometry

For live staining, cells were incubated with BODIPY 493/503 (ThermoFisher, 0.2 μg/mL) for 10 min at RT, MitoTracker Deep Red (Invitrogen M22426, 2 nM) for 15 min at RT, or CellROX Deep Red (ThermoFisher C10422, 2 μM) for 20 min at 37°C. The cells were dissociated by trypsin digestion, washed, and resuspended in PBS 1×. GFP was monitored in order to evaluate the reprogramming efficiency in icMEFs. For intracellular staining, iCMs on D11/12 were harvested by trypsin digestion, washed with ice‐cold FACS buffer (DPBS supplemented with 2% FBS and 2 mM EDTA), fixed, and stained using Cell Fixation and Permeabilization Kits (BD Biosciences). The antibodies were performed for 1 h at RT. The following antibodies were used: primary anti‐mouse cardiac Troponin T (Invitrogen, MA5‐12960, 1:200), anti‐mouse Tropomyosin Alexa 488 conjugated (Santa Cruz Biotechnology, 1:200), and goat anti‐mouse Alexa 488 (Invitrogen A11001, 1:600). For cell cycle analysis, the cells were collected, washed with PBS 1× supplemented with FBS (2%), and centrifuged at 300 ***g*** for 5 min at RT. Cold ethanol (70%) was added dropwise and vortexed. Cells were fixed for 1 h at 4°C, washed, and resuspended in the Propidium Iodide Staining Solution (SIGMA, 2 μg/mL) with RNAse A (1000 μg/mL, Thermo Scientific) and incubated overnight at 4°C. Cells were analyzed on a BD Accuri™ C6 Flow Cytometer (BD Biosciences) and FlowJo software (RRID: SCR_008520). Around 20.000 and between 8.000–17.000 events (alive/count) were acquired for live and intracellular staining analyses, respectively.

### 4.6 RNA extraction and RT‐qPCR

For RNA analysis, total RNA was extracted from cells using either the NZYol (NZYTech) or GRS Total RNA Kit (GRiSP) according to the manufacturer's instructions and reverse transcribed using NZY First‐Strand cDNA Synthesis (NZYtech) according to the manufacturer's instructions. Quantitative real‐time PCR (qPCR) was performed using NZYSupreme qPCR Green Master Mix (2×), (NZYTech) in an ABI 7500 thermocycler. The $2^{-\Delta \Delta C_{t}}$ method was used to determine gene expression levels. All primer sequences are listed in Table S6.

### 4.7 ChIP‐qPCR

ChIP was performed using the MAGnify™ Chromatin Immunoprecipitation System (ThermoFisher, 492,024), according to manufacturers' instructions. Briefly, 1.17 × 10^6^ cells were crosslinked with 1% formaldehyde at RT for 10 min and sheared on ice using a Clifton SW3H water bath sonicator (60 min, uninterrupted). For each ChIP, diluted chromatin from approximately 2 × 10^5^ cells was incubated with rabbit anti‐H3K4me3 (3 μg, Active Motif, 39,915), rabbit anti‐H3K27me3 (5 μg Active Motif, 39,155), rabbit anti‐H3K9me1 (4 μg, Abcam, ab176880), rabbit anti‐H4K20me2 (4 μg, Abcam, ab9052), or rabbit anti‐IgG (1 μg), coupled to Dynabeads™ Protein A/G beads, overnight at 4°C. After washing, the chromatin and DNA were reverse‐crosslinked, and the DNA was purified. qPCR was performed using NZYSupreme qPCR Green Master Mix (2x) (NZYtech, MB41903) on a CFX Connect Real‐Time PCR System (Bio‐Rad). ChIP‐qPCR primers were used as described previously (Zhou et al., 2017) and are listed in Table S6.

### 4.8 Immunofluorescence

Cells were dissociated, counted, and plated at a density of 10.4 × 10^3^ cells per well in 8‐well ibidi plates (ibidi 81,506) or at 2 × 10^4^ cells per well in 24‐well plates with glass coverslips previously coated with 0.1% gelatin porcine (SIGMA) and incubated at 37°C with 5% CO_2_, until the next day. Cells were fixed with PFA (3.7%, Thermo Scientific), permeabilized with 0.1% Triton X‐100 (SIGMA), and blocked with 1% BSA (SIGMA) in PBS 1× for 1 h at RT. Primary anti‐mouse HSP‐60 (BD Biosciences, 1:200), anti‐rabbit TOM20 (Proteintech, 1:100), anti‐mouse cardiac Troponin T (Abcam, 1:150), anti‐rabbit Connexin‐43 (SIGMA, 1:200), or anti‐mouse α‐actinin sarcomeric (SIGMA, 1:300) antibodies were incubated at 4°C overnight followed by secondary antibodies anti‐rabbit Alexa 488 (Invitrogen, 1:500), anti‐mouse Alexa 488 (Invitrogen, 1:500), anti‐mouse Alexa 594 (Invitrogen, 1:500), or α‐GFP Alexa 488 (Invitrogen, 1:500) for 1 h at RT. CellROX Deep Red (ThermoFisher C10422, 5 μM) and MitoTracker Red (Invitrogen M7512, 150 nM) staining was performed directly on the plates in HBSS 1× (Gibco) for 30 min at 37°C in the dark. For immunostaining combined with MitoTracker Red, the cells were fixed with 3.7% PFA, permeabilized with 0.5% Triton X‐100 (SIGMA), and blocked with 5% goat serum (Abbkine Scientific) and 0.3% Triton X‐100 in PBS 1× for 1 h at RT with agitation. Primary rabbit anti‐LC3 (Cell Signaling #3868, 1:600) antibody was incubated at 4°C overnight followed by secondary antibody anti‐rabbit Alexa 488 (ThermoFisher Scientific, 1:600) for 1 h at RT. Cells were mounted using Vectashield with DAPI (vectorlabs). For TMRE live cell imaging, FCCP (Abcam, 20 μM) was added for 10 min at 37°C for negative control. TMRE red (Abcam, ab113852, 50 nM) prepared in growth medium was added for 20 min at 37°C in the dark, and the cells were washed and imaged. Ca^2+^ signals were imaged using a Rhod‐3 Calcium Imaging Kit (Life Technologies) according to the manufacturer's instructions. Briefly, cells were loaded with Rhod‐3 for 1 h at RT, washed, and incubated with 2.5 mM probenecid in HBSS for an additional 1 h to allow de‐esterification, and Rhod‐3‐labeled cells were analyzed at RT.

### 4.9 Statistical analysis

Graphical data denote the mean ± s.d or mean ± s.e.m. (of *n* = 3 or more biological replicates, cells, or mice) and are depicted by column graph scatter dot plot using GraphPad Prism 8.4.0 software (SCR_002798) or as otherwise specified. Details of the sample numbers can be found in the appropriate figure legend. Unless indicated otherwise, the *p* values for analyses was determined by two‐tailed Student's *t*‐test between two groups and one‐way analysis of variance (ANOVA) followed by Tukey's post‐test for multiple comparisons or Kruskal Wallis with a false discovery rate correction (with an FDR value of 0.05). For normality, the Shapiro–Wilk test was performed. *p < 0.05; **p < 0.01; ***p < 0.001; ****p < 0.0001.

## AUTHOR CONTRIBUTIONS

F.S. and M.C. developed the experimental approaches and methodology and performed the majority of the experiments and data analysis. R.D., and B.B. performed experiments and data analysis. R.S.F. established the methodology for mitochondria network analysis. D. T. performed the calcium imaging. R.N. and T.B. performed the LC‐MS‐based histone proteomics. P.D. performed the LC‐MS‐based metabolomics. J.T., P.O., C.B., and B.B. de J. performed experiments, provided tools, or analyzed the data. C.B, B.B. de J., and S.N.‐P. acquired funding: S.N.‐P. conceptualized and administered the project, and wrote the manuscript with the help of all authors.

## CONFLICT OF INTEREST STATEMENT

The authors declare that they have no conflict of interest.

## Supporting information


Appendix S1.

Movie S1.

Movie S2.

Movie S3.

## Data Availability

RNA‐seq, LC–MS‐based metabolomics and LC‐MS‐based histone proteomics data, uncropped immunoblots, and additional experimental procedures from this work are available at Appendix S1, in Tables S1–S5 and Figure S6. The histone proteomics data was deposited in the ProteomeXchange Consortium PRIDE partner repository with the dataset identifier PXD046542, and the RNA‐seq data in the European Nucleotide Archive with the dataset identifier Project PRJEB77697 and Study ERP162057. MS‐based metabolomics data is available upon request. This study does not report original code. Any additional information required to reanalyze the data reported in this paper is available from the lead contact upon request.

## References

[acel14371-bib-0001] Addis, R. C. , Ifkovits, J. L. , Pinto, F. , Kellam, L. D. , Esteso, P. , Rentschler, S. , Christoforou, N. , Epstein, J. A. , & Gearhart, J. D. (2013). Optimization of direct fibroblast reprogramming to cardiomyocytes using calcium activity as a functional measure of success. Journal of Molecular and Cellular Cardiology, 60, 97–106. 23591016 10.1016/j.yjmcc.2013.04.004 PMC3679282

[acel14371-bib-0002] Bachamanda Somesh, D. , Klose, K. , Maring, J. A. , Kunkel, D. , Jürchott, K. , Protze, S. I. , Klein, O. , Nebrich, G. , Becker, M. , Krüger, U. , Nazari‐Shafti, T. Z. , Falk, V. , Kurtz, A. , Gossen, M. , & Stamm, C. (2023). Cardiomyocyte precursors generated by direct reprogramming and molecular beacon selection attenuate ventricular remodeling after experimental myocardial infarction. Stem Cell Research & Therapy, 14, 296. 10.1186/s13287-023-03519-w 37840130 PMC10577947

[acel14371-bib-0003] Bernardes de Jesus, B. , Marinho, S. P. , Barros, S. , Sousa‐Franco, A. , Alves‐Vale, C. , Carvalho, T. , & Carmo‐Fonseca, M. (2018). Silencing of the lncRNA Zeb2‐NAT facilitates reprogramming of aged fibroblasts and safeguards stem cell pluripotency. Nature Communications, 9, 94. 10.1038/s41467-017-01921-6 PMC5758807 29311544

[acel14371-bib-0004] Cardoso, A. C. , Lam, N. T. , Savla, J. J. , Nakada, Y. , Pereira, A. H. M. , Elnwasany, A. , Menendez‐Montes, I. , Ensley, E. L. , Bezan Petric, U. , Sharma, G. , Sherry, A. D. , Malloy, C. R. , Khemtong, C. , Kinter, M. T. , Tan, W. L. W. , Anene‐Nzelu, C. G. , Foo, R. S. Y. , Nguyen, N. U. N. , Li, S. , … Sadek, H. A. (2020). Mitochondrial substrate utilization regulates cardiomyocyte cell‐cycle progression. Nature Metabolism, 2, 167–178. PMC7331943 32617517

[acel14371-bib-0005] Carrer, A. , Parris, J. L. D. , Trefely, S. , Henry, R. A. , Montgomery, D. C. , Torres, A. , Viola, J. M. , Kuo, Y.‐M. , Blair, I. A. , Meier, J. L. , Andrews, A. J. , Snyder, N. W. , & Wellen, K. E. (2017). Impact of a high‐fat diet on tissue acyl‐CoA and histone acetylation levels. The Journal of Biological Chemistry, 292, 3312–3322. 28077572 10.1074/jbc.M116.750620 PMC5336165

[acel14371-bib-0006] Chen, J. X. , Krane, M. , Deutsch, M. A. , Wang, L. , Rav‐Acha, M. , Gregoire, S. , Engels, M. C. , Rajarajan, K. , Karra, R. , Abel, E. D. , Wu, J. C. , Milan, D. , & Wu, S. M. (2012). Inefficient reprogramming of fibroblasts into cardiomyocytes using Gata4, Mef2c, and Tbx5. Circulation Research, 111, 50–55. 22581928 10.1161/CIRCRESAHA.112.270264 PMC3390172

[acel14371-bib-0007] Christoforou, N. , Chellappan, M. , Adler, A. F. , Kirkton, R. D. , Wu, T. , Addis, R. C. , Bursac, N. , & Leong, K. W. (2013). Transcription factors MYOCD, SRF, Mesp1 and SMARCD3 enhance the cardio‐inducing effect of GATA4, TBX5, and MEF2C during direct cellular reprogramming. PLoS One, 8, e63577. 23704920 10.1371/journal.pone.0063577 PMC3660533

[acel14371-bib-0008] Claycomb, W. C. , Lanson, N. A. J. , Stallworth, B. S. , Egeland, D. B. , Delcarpio, J. B. , Bahinski, A. , & Izzo, N. J. J. (1998). HL‐1 cells: A cardiac muscle cell line that contracts and retains phenotypic characteristics of the adult cardiomyocyte. Proceedings of the National Academy of Sciences of the United States of America, 95, 2979–2984. 9501201 10.1073/pnas.95.6.2979 PMC19680

[acel14371-bib-0009] Cornacchia, D. , Zhang, C. , Zimmer, B. , Chung, S. Y. , Fan, Y. , Soliman, M. A. , Tchieu, J. , Chambers, S. M. , Shah, H. , Paull, D. , Konrad, C. , Vincendeau, M. , Noggle, S. A. , Manfredi, G. , Finley, L. W. S. , Cross, J. R. , Betel, D. , & Studer, L. (2019). Lipid deprivation induces a stable, naive‐to‐primed intermediate state of pluripotency in human PSCs. Cell Stem Cell, 25, 120–136.e10. 10.1016/j.stem.2019.05.001 31155483 PMC7549840

[acel14371-bib-0010] Gibb, A. A. , Lazaropoulos, M. P. , & Elrod, J. W. (2020). Myofibroblasts and fibrosis. Circulation Research, 127, 427–447. 32673537 10.1161/CIRCRESAHA.120.316958 PMC7982967

[acel14371-bib-0011] Gong, G. , Song, M. , Csordas, G. , Kelly, D. P. , Matkovich, S. J. , & Dorn, G. W., 2nd . (2015). Parkin‐mediated mitophagy directs perinatal cardiac metabolic maturation in mice. Science, 350, aad2459. 26785495 10.1126/science.aad2459 PMC4747105

[acel14371-bib-0012] Hashimoto, H. , Olson, E. N. , & Bassel‐Duby, R. (2018). Therapeutic approaches for cardiac regeneration and repair. Nature Reviews Cardiology, 15, 585–600. 10.1038/s41569-018-0036-6 29872165 PMC6241533

[acel14371-bib-0013] Hirai, H. , & Kikyo, N. (2014). Inhibitors of suppressive histone modification promote direct reprogramming of fibroblasts to cardiomyocyte‐like cells. Cardiovascular Research, 102, 189–190. 10.1093/cvr/cvu023 PMC3958621 24477643

[acel14371-bib-0014] Hong, X. , Isern, J. , Campanario, S. , Perdiguero, E. , Ramírez‐Pardo, I. , Segalés, J. , Hernansanz‐Agustín, P. , Curtabbi, A. , Deryagin, O. , Pollán, A. , González‐Reyes, J. A. , Villalba, J. M. , Sandri, M. , Serrano, A. L. , Enríquez, J. A. , & Muñoz‐Cánoves, P. (2022). Mitochondrial dynamics maintain muscle stem cell regenerative competence throughout adult life by regulating metabolism and mitophagy. Cell Stem Cell, 29, 1298–1314.e10. 35998641 10.1016/j.stem.2022.07.009

[acel14371-bib-0015] Ieda, M. , Fu, J. , Delgado‐olguin, P. , Vedantham, V. , Hayashi, Y. , Bruneau, B. G. , & Srivastava, D. (2010). Direct reprogramming of fibroblasts into functional cardiomyocytes by defined factors. Cell, 142, 375–386. 20691899 10.1016/j.cell.2010.07.002 PMC2919844

[acel14371-bib-0016] Intlekofer, A. M. , & Finley, L. W. S. (2019). Metabolic signatures of cancer cells and stem cells. Nature Metabolism, 1, 177–188. 10.1038/s42255-019-0032-0 PMC6594714 31245788

[acel14371-bib-0017] Kojima, H. , & Ieda, M. (2017). Discovery and progress of direct cardiac reprogramming. Cellular and Molecular Life Sciences, 74, 2203–2215. 28197667 10.1007/s00018-017-2466-4 PMC11107684

[acel14371-bib-0018] Kurotsu, S. , Sadahiro, T. , Fujita, R. , Tani, H. , Yamakawa, H. , Tamura, F. , Isomi, M. , Kojima, H. , Yamada, Y. , Abe, Y. , Murakata, Y. , Akiyama, T. , Muraoka, N. , Harada, I. , Suzuki, T. , Fukuda, K. , & Ieda, M. (2020). Soft matrix promotes cardiac reprogramming via inhibition of YAP/TAZ and suppression of fibroblast signatures. Stem Cell Reports, 15, 612–628. 32857980 10.1016/j.stemcr.2020.07.022 PMC7486305

[acel14371-bib-0019] Lehman, J. J. , & Kelly, D. P. (2002). Transcriptional activation of energy metabolic switches in the developing and hypertrophied heart. Clinical and Experimental Pharmacology & Physiology, 29, 339–345. 11985547 10.1046/j.1440-1681.2002.03655.x

[acel14371-bib-0020] Li, X. , Wu, F. , Günther, S. , Looso, M. , Kuenne, C. , Zhang, T. , Wiesnet, M. , Klatt, S. , Zukunft, S. , Fleming, I. , Poschet, G. , Wietelmann, A. , Atzberger, A. , Potente, M. , Yuan, X. , & Braun, T. (2023). Inhibition of fatty acid oxidation enables heart regeneration in adult mice. Nature, 622, 619–626. 37758950 10.1038/s41586-023-06585-5 PMC10584682

[acel14371-bib-0021] Liu, Z. , Chen, O. , Zheng, M. , Wang, L. , Zhou, Y. , Yin, C. , Liu, J. , & Qian, L. (2016). Re‐patterning of H3K27me3, H3K4me3 and DNA methylation during fibroblast conversion into induced cardiomyocytes. Stem Cell Research, 16, 507–518. 26957038 10.1016/j.scr.2016.02.037 PMC4828257

[acel14371-bib-0022] Lopaschuk, G. D. , Collins‐Nakai, R. L. , & Itoi, T. (1992). Developmental changes in energy substrate use by the heart. Cardiovascular Research, 26, 1172–1180. 10.1093/cvr/26.12.1172 1288863

[acel14371-bib-0023] Maryanovich, M. , & Gross, A. (2013). A ROS rheostat for cell fate regulation. Trends in Cell Biology, 23, 129–134. 10.1016/j.tcb.2012.09.007 23117019

[acel14371-bib-0024] Menendez‐Montes, I. , Escobar, B. , Palacios, B. , Gómez, M. J. , Izquierdo‐Garcia, J. L. , Flores, L. , Jiménez‐Borreguero, L. J. , Aragones, J. , Ruiz‐Cabello, J. , Torres, M. , & Martin‐Puig, S. (2016). Myocardial VHL‐HIF signaling controls an embryonic metabolic switch essential for cardiac maturation. Developmental Cell, 39, 724–739. 27997827 10.1016/j.devcel.2016.11.012

[acel14371-bib-0025] Muraoka, N. , Yamakawa, H. , Miyamoto, K. , Sadahiro, T. , Umei, T. , Isomi, M. , Nakashima, H. , Akiyama, M. , Wada, R. , Inagawa, K. , Nishiyama, T. , Kaneda, R. , Fukuda, T. , Takeda, S. , Tohyama, S. , Hashimoto, H. , Kawamura, Y. , Goshima, N. , Aeba, R. , … Ieda, M. (2014). MiR‐133 promotes cardiac reprogramming by directly repressing Snai1 and silencing fibroblast signatures. The EMBO Journal, 33, 1565–1581. 10.15252/embj.201387605 24920580 PMC4198052

[acel14371-bib-0026] Pal, S. , & Tyler, J. K. (2016). Epigenetics and aging. Science Advances, 2, e1600584. 10.1126/sciadv.1600584 27482540 PMC4966880

[acel14371-bib-0027] Paquin, K. L. , & Howlett, N. G. (2018). Understanding the histone DNA repair code: H4K20me2 makes its mark. Molecular Cancer Research, 16, 1335–1345. 10.1158/1541-7786.MCR-17-0688 29858375 PMC7083049

[acel14371-bib-0028] Qian, L. , Huang, Y. , Spencer, C. I. , Foley, A. , Vedantham, V. , Liu, L. , Conway, S. J. , Fu, J. D. , & Srivastava, D. (2012). In vivo reprogramming of murine cardiac fibroblasts into induced cardiomyocytes. Nature, 485, 593–598. 22522929 10.1038/nature11044 PMC3369107

[acel14371-bib-0029] Singh, V. P. , Pinnamaneni, J. P. , Pugazenthi, A. , Sanagasetti, D. , Mathison, M. , Wang, K. , Yang, J. , & Rosengart, T. K. (2020). Enhanced generation of induced cardiomyocytes using a small‐molecule cocktail to overcome barriers to cardiac cellular reprogramming. Journal of the American Heart Association, 9, e015686. 32500803 10.1161/JAHA.119.015686 PMC7429035

[acel14371-bib-0030] Song, K. , Nam, Y. J. , Luo, X. , Qi, X. , Tan, W. , Huang, G. N. , Acharya, A. , Smith, C. L. , Tallquist, M. D. , Neilson, E. G. , Hill, J. A. , Bassel‐Duby, R. , & Olson, E. N. (2012). Heart repair by reprogramming non‐myocytes with cardiac transcription factors. Nature, 485, 599–604. 10.1038/nature11139 22660318 PMC3367390

[acel14371-bib-0031] Soonpaa, M. H. , Kim, K. K. , Pajak, L. , Franklin, M. , & Field, L. J. (1996). Cardiomyocyte DNA synthesis and binucleation during murine development. The American Journal of Physiology, 271, H2183–H2189. 8945939 10.1152/ajpheart.1996.271.5.H2183

[acel14371-bib-0032] Stone, N. J. , Levy, R. I. , Fredrickson, D. S. , & Verter, J. (1974). Coronary artery disease in 116 kindred with familial type II hyperlipoproteinemia. Circulation, 49, 476–488. 4813182 10.1161/01.cir.49.3.476

[acel14371-bib-0033] Vaseghi, H. R. , Yin, C. , Zhou, Y. , Wang, L. , Liu, J. , & Qian, L. (2016). Generation of an inducible fibroblast cell line for studying direct cardiac reprogramming. Genesis, 54, 398–406. 27194122 10.1002/dvg.22947 PMC4956550

[acel14371-bib-0034] Wada, R. , Muraoka, N. , Inagawa, K. , Yamakawa, H. , Miyamoto, K. , Sadahiro, T. , Umei, T. , Kaneda, R. , Suzuki, T. , Kamiya, K. , Tohyama, S. , Yuasa, S. , Kokaji, K. , Aeba, R. , Yozu, R. , Yamagishi, H. , Kitamura, T. , Fukuda, K. , & Ieda, M. (2013). Induction of human cardiomyocyte‐like cells from fibroblasts by defined factors. Proceedings of the National Academy of Sciences, 110, 12667–12672. 10.1073/pnas.1304053110 PMC3732928 23861494

[acel14371-bib-0035] Wang, H. , Yang, Y. , Liu, J. , & Qian, L. (2021). Direct cell reprogramming: Approaches, mechanisms and progress. Nature Reviews Molecular Cell Biology, 22, 410–424. 10.1038/s41580-021-00335-z 33619373 PMC8161510

[acel14371-bib-0036] Wang, L. , Liu, Z. , Yin, C. , Asfour, H. , Chen, O. , Li, Y. , Bursac, N. , Liu, J. , & Qian, L. (2015). Stoichiometry of Gata4, Mef2c, and Tbx5 influences the efficiency and quality of induced cardiac myocyte reprogramming. Circulation Research, 116, 237–244. 25416133 10.1161/CIRCRESAHA.116.305547 PMC4299697

[acel14371-bib-0037] Wang, L. , Ma, H. , Huang, P. , Xie, Y. , Near, D. , Wang, H. , Xu, J. , Yang, Y. , Xu, Y. , Garbutt, T. , Zhou, Y. , Liu, Z. , Yin, C. , Bressan, M. , Taylor, J. M. , Liu, J. , & Qian, L. (2020). Down‐regulation of Beclin1 promotes direct cardiac reprogramming. Science Translational Medicine, 12, 1–23. 10.1126/scitranslmed.aay7856 PMC8188650 33087505

[acel14371-bib-0038] Yucel, N. , Wang, Y. X. , Mai, T. , Porpiglia, E. , Lund, P. J. , Markov, G. , Garcia, B. A. , Bendall, S. C. , Angelo, M. , & Blau, H. M. (2019). Glucose metabolism drives histone acetylation landscape transitions that dictate muscle stem cell function. Cell Reports, 27, 3939–3955.e6. 31242425 10.1016/j.celrep.2019.05.092 PMC6788807

[acel14371-bib-0039] Zhou, Y. , Wang, L. , Liu, Z. , Alimohamadi, S. , Yin, C. , Liu, J. , & Qian, L. (2017). Comparative gene expression analyses reveal distinct molecular signatures between differentially reprogrammed cardiomyocytes. Cell Reports, 20, 3014–3024. 28954220 10.1016/j.celrep.2017.09.005 PMC5659840

