# Supplementary material for: Age‐associated metabolic and epigenetic barriers during direct reprogramming of mouse fibroblasts into induced cardiomyocytes
Source: Aging Cell. 2024 Nov 14;24(2):e14371. doi: 10.1111/acel.14371 (PMC11822649; doi:10.1111/acel.14371)
Supplement: Supplementary file 1 — Appendix S1. [file ACEL-24-e14371-s002.zip › Figure captions.docx]

FIGURE S1. Transdifferentiation of mouse fibroblasts into iCMs is accompanied by epigenetic transitions. (a-c) qPCR analysis of the relative expression of the indicated genes in parental MEFs, AEFs and old AEFs (a), mock or MGT retroviral-transduced at D11/12 (b, c) (fold to MEFs mock). (d-f) Bulk RNA-sequencing analysis in mock and MGT-transduced MEFs and AEFs at D11, and HL-1 mouse cardiomyocyte cell line. Correlation heatmap (d) and Principal component analysis (PCA) with a scatterplot of PC1 versus PC2 (e) of the indicated samples. Differentially expressed genes (DEGs) displaying number for all, down- and up- regulated between the display set of conditions (f). (g-j) Mass spectrometry-based histone acetylation and methylation proteomic bulk analysis in mock and MGT-transduced MEFs and AEFs at D11, and HL-1 mouse cardiomyocyte cell line. Principal component analysis (PCA) based on histone PTM data obtained from the z scores of the samples shown in Figure 2a (g). Selected histone modified peptides showing the levels of tri-methylated form of H3 lysine 27 and mono-methylated lysine 36 (h), tri-methylated form of H3 lysine 4 (i) and mono-methylated form of H3 lysine 27 (j), the bar indicates a p-value <0.05. (k-m) ChIP-qPCR for H3K27me3 (k), H3K9me1 (l) and H4K20me2 (m) for fibroblast genes (*Col3a1*, *Postn*) on mock and MGT-transduced MEFs and AEFs on D11 expressed as % to Input and comparisons to corresponding mock for each gene. (n) Bulk RNA-sequencing difference analysis (without log2fc and FDR filtering) for the expression amount of *Kmt5b* and *Setdb1* in mock and MGT-transduced MEFs and AEFs at D11. Each point on the plot indicates individual measurements and mean ± s.d of *n* = 3–7 biological replicates from one representative experiment or three independent experiments. p values were calculated by one-way ANOVA with False Discovery Rate followed by Tukey's post-test for multiple comparisons. **p* < 0.05; ***p* < 0.01; ****p* < 0.001; *****p* < 0.0001. Data analysis and visualization were performed using GraphPad Prism (RRID:SCR_002798), Perseus (RRID:SCR_015753) (g-j), R project for statistical computing (RRID:SCR_001905) prcomp (d) and pheatmap (e).

FIGURE S2. Transdifferentiation of mouse fibroblasts into iCMs is accompanied by metabolic and bioenergetic transitions resembling those present in cardiomyocytes. (a-d) LC–MS-based untargeted metabolomics bulk analysis of MEFs mock or MGT-transduced at D11, and the HL-1 mouse cardiomyocyte cell line. Selected box-plot analysis relative abundances of the identified metabolites phenylethylamine (a), L-glutamine (b) and adenine (c) between HL-1 (green boxplots), MEFs mock (orange boxplots) and MEFs MGT (purple boxplots). Dot plot of identified metabolomic pathways in MEFs mock and MGT displaying log(p) values from pathway enrichment analysis vs. values of the pathways impact, where higher impact values represent the relative importance of the pathway, the size of the circle indicates the impact of the pathway while the color represents the significance (d). (e) Cell cycle analysis of propidium iodide staining by flow cytometry displaying the percentage of cells in each phase (sub-G1, G0/G1, S and G2/M) in MEFs and AEFs mock or MGT transduced at D11. (f-h) Oxygen consumption rate (OCR) measurement in MEFs and AEFs mock or MGT transduced or HL-1 at D11 using Seahorse XF96 Cell Mito Stress Test (f), basal extracellular acidification rate (ECAR) (g) and OCR/ECAR ratio (h). Data were normalized to cell mass using the sulforhodamine B (SRB) assay. Each point on the plot indicates individual measurements and mean ± s.d of *n* = 3–7 biological replicates from one representative experiment (a-e) or *n* = 26–28 technical replicates from three independent experiments (f-h). p values were calculated by one-way ANOVA and Kruskal-Wallis tests with False Discovery Rate multiple comparisons test by post-hoc Tukey's or Dunn's test. ****p* < 0.001; *****p* < 0.0001. Data analysis and visualization were performed using GraphPad Prism (RRID:SCR_002798), R package ggplot2 (RRID:SCR_014601) (a-c) and MetaboAnalyst 5.0 (RRID:SCR_015539) (d).

FIGURE S3. Extensive remodeling of mitochondrial network and mitophagy takes place during direct cardiac conversion. (a-b) Analysis of the number of TOM20 particles (a) and total area (b) in MEFs and AEFs mock or MGT-transduced at D11 or HL-1 cells. (c) Quantification of immunofluorescence staining for cTNT per cell (integrated density, IntDen) in MEFs and AEFs mock or MGT-transduced at D11. (d) Analysis of TOM20 branch junctions per mitochondria in MGT-transduced cTNT positive and negative cells (20 cTNT^+^/36 total cells for MEFs MGT and 6 cTNT^+^/22 total cells for AEFs MGT) or mock cells at D11. (e) Flow cytometry quantification of BODIPY 493/503 depicted as relative median fluorescence intensity (MFI, fold to corresponding mock) in MEFs and AEFs mock or MGT-transduced at D11. (f-f’) Immunoblotting analysis for PGC-1α and β-actin in whole cell extracts depicting densitometric quantification (f, normalized to β-actin) and representative images (f’, uncropped images in Figure S6) MEFs and AEFs mock or MGT-transduced at D11. (g) Densitometric quantification of immunoblotting analysis for p62 and PINK1 normalized to β-actin (related to Figure 4j) in MEFs and AEFs mock or MGT-transduced at D11. (h) Representative flow cytometry plots of cardiac troponin (cTnT) in MEFs and AEFs mock or MGT retroviral-infected on D11, untreated or supplemented with rapamycin (Rap, 10 nM) or urolithin A (UroA, 5 µM) (related to Figure 4k).
Each point on the plot indicates individual measurements and mean ± s.d of *n* = 6–43 (a-d) cells analyzed or *n* = 3–5 biological replicates from one representative or two independent experiments. p values were calculated by Kruskal Wallis with false discovery rate correction and one-way ANOVA and Tukey's post-test for multiple comparisons. **p* < 0.05 between the indicated groups. Graphs were created using GraphPad Prism (RRID:SCR_002798) and flow cytometry plots (h) with FlowJo (RRID:SCR_008520).

FIGURE S4. Metabolic modulation can bypass epigenetic and age-associated barriers to DCC. (a) Representative images of α-MHC-GFP, mitochondria HSP-60 and DAPI in icMEFs with no Dox or Dox for 3 days. (b-d) Analysis of HSP-60 fluorescence corrected by cell area (b), form factor (shape, c) and branches per mitochondria (network connectivity, d) in icMEFs with no Dox or Dox (GFP-expressing cells) for 3 days. (e) Flow cytometry quantification of CellROX (ROS) depicted as the fold change of the relative median fluorescence intensity (MFI) in icMEFs with no Dox or Dox for 3 days and in the presence of Resveratrol (RVT, 20 nM). (f-l) Mass spectrometry-based histone acetylation and methylation proteomic bulk analysis in neonatal mouse cardiac myocytes (NMCM) and fibroblasts (NMCF) and MGT-transduced MEFs and AEFs at D11 in the presence of standard medium (S: glucose 25 mM, FBS 10%) only or supplemented with sodium acetate and α-KG (S*: sodium acetate 5 mM and α-KG 1,5 mM), low glucose (LG: glucose 1 mM, FBS 10%) or low lipids (LL: glucose 25 mM, FBS 1%) growth medium. PCA based on histone PTM data obtained from the z scores of the samples shown in the heatmap display of histone PTM levels for MGT-transduced MEFs (f) and AEFs (g). Levels of unmodified form of H3 lysine 4 in the indicated samples, the bar indicates a p-value <0.05 (h). Heatmap display of histone PTM levels in AEFs samples, NMCM and NMCF clustered based on Pearson's correlation, grey squares indicates peptides that were not quantified. The panel on the right shows significant changes (grey) between NMCM and the indicated comparisons (i). Display of selected histone modified peptides showing the levels of mono-methylated form of H3 lysine 9 (j), bi-acetylated form of H4 lysine 4–17 (k) and mono-methylated form of H3 lysine 9 and mono-acetylated lysine 14 (l) in the indicated samples, the bar indicates a p-value <0.05. Each point on the plot indicates individual measurements and mean ± s.d of *n* = 9–10 cells analyzed (b-d) or *n* = 3–5 biological replicates from two independent or one representative experiment. p values were calculated by unpaired t test or one-way ANOVA or Kruskal Wallis with False Discovery Rate correction by post-hoc Tukey's test. **p* < 0.05; ***p* < 0.01 . Graphs were created using GraphPad Prism (RRID:SCR_002798) and Perseus (RRID:SCR_015753) (f-l). Scale bar, 20 µm in (a).

FIGURE S5. Dietary lipids improve DCC of adult fibroblasts ex vivo. (a-b) qPCR analysis of the relative expression of the indicated genes in CFs (a) and AEFs (b) isolated from animals under CD, HFD and LFD. (c-d) Densitometric quantification of immunoblotting analysis for total histone H3 (c) and H3 pan-acetylated (acetyl K9 + K14 + K18 + K23 + K27) (d) normalized to Ponceau S staining (fold), from whole cell extracts of CFs isolated from animals under CD, HFD and LFD. (e) qPCR analysis of the relative expression of the indicated genes in AEFs isolated from animals under CD, HFD and LFD subjected to mock or MGT transduction at D11/12 (fold to corresponding mock). (f) TMRE quantification depicted as relative mean fluorescence intensity in AEFs isolated from animals under CD, HFD and LFD subjected to mock or MGT transduction at D11. Each point on the plot indicates individual measurements and mean ± s.e.m. of *n* = 3–9 biological replicates from one representative or two independent experiments. p values were calculated by one-way ANOVA with False Discovery Rate followed by Tukey's post-test for multiple comparisons or Kruskal-Wallis test. **p* < 0.05; ***p* < 0.01; ****p* < 0.001; *****p* < 0.0001. Graphs were created using GraphPad Prism (RRID:SCR_002798).

FIGURE S6. Uncropped membranes relative to the immunoblotting for PGC-1α, p62, PINK1 and β-actin displayed in Figure S3f’ and Figure 4j and total Histone H3, H3 pan-acetylated (acetyl K9 + K14 + K18 + K23 + K27) or Ponceau S displayed in Figure 6c’.
