# Supplementary material for: Age‐associated metabolic and epigenetic barriers during direct reprogramming of mouse fibroblasts into induced cardiomyocytes
Source: Aging Cell. 2024 Nov 14;24(2):e14371. doi: 10.1111/acel.14371 (PMC11822649; doi:10.1111/acel.14371)

Santos *et al.*, FIGURE S6

PGC-1α

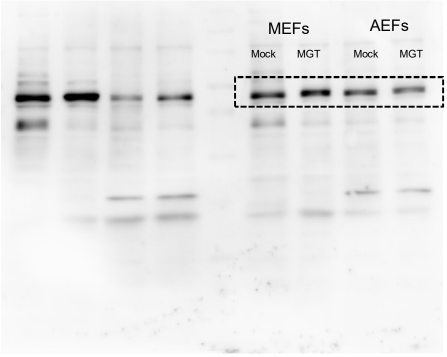

p62

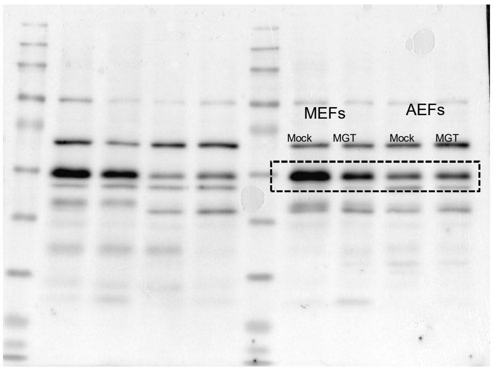

β-actin

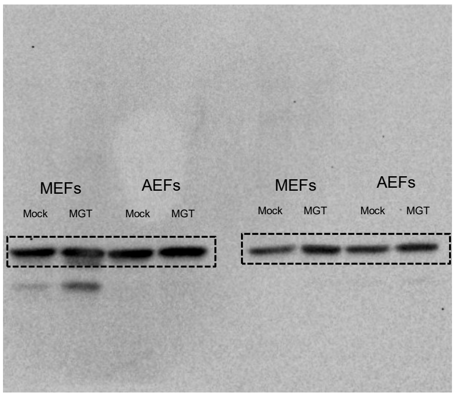

PINK1

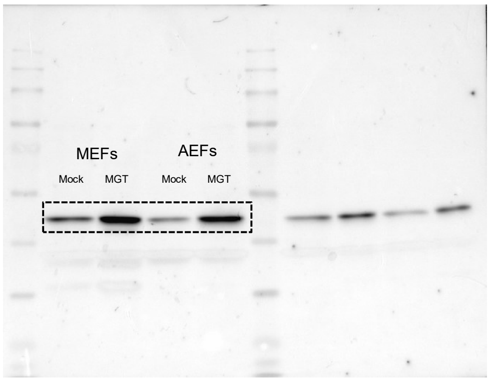

β-actin

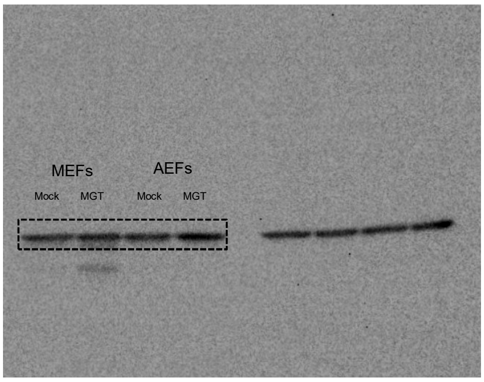

Ponceau S (Histone H3)

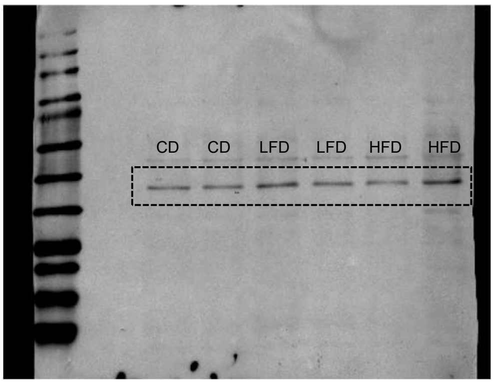

Ponceau S (H3ac pan-acetyl) pan)

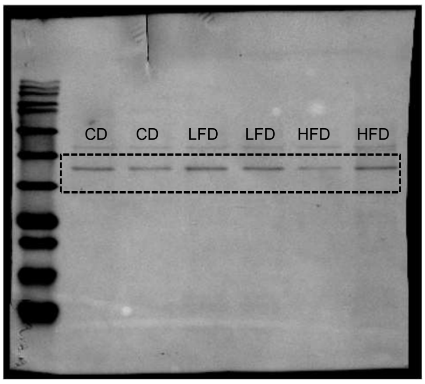

Histone H3

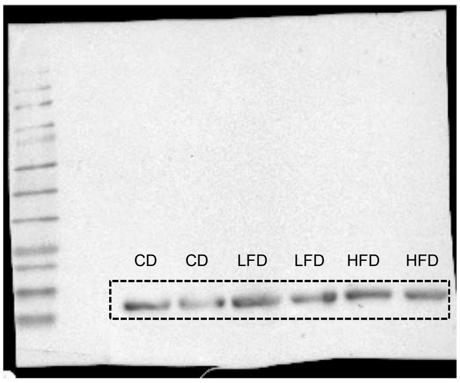

H3ac pan-acetyl

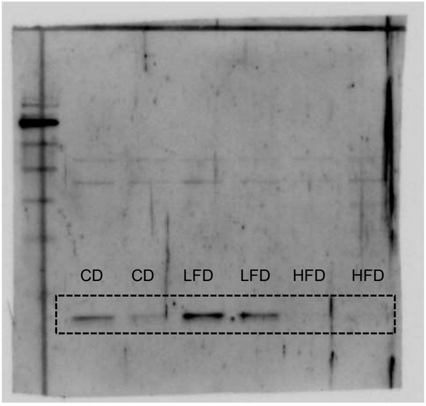

Supplement: Supplementary file 1 — Appendix S1. [file ACEL-24-e14371-s002.zip › FigS6 Santos et al.pdf]
