# Supplementary figures and images for: Age‐associated metabolic and epigenetic barriers during direct reprogramming of mouse fibroblasts into induced cardiomyocytes
Source: Aging Cell. 2024 Nov 14;24(2):e14371. doi: 10.1111/acel.14371 (PMC11822649; doi:10.1111/acel.14371)

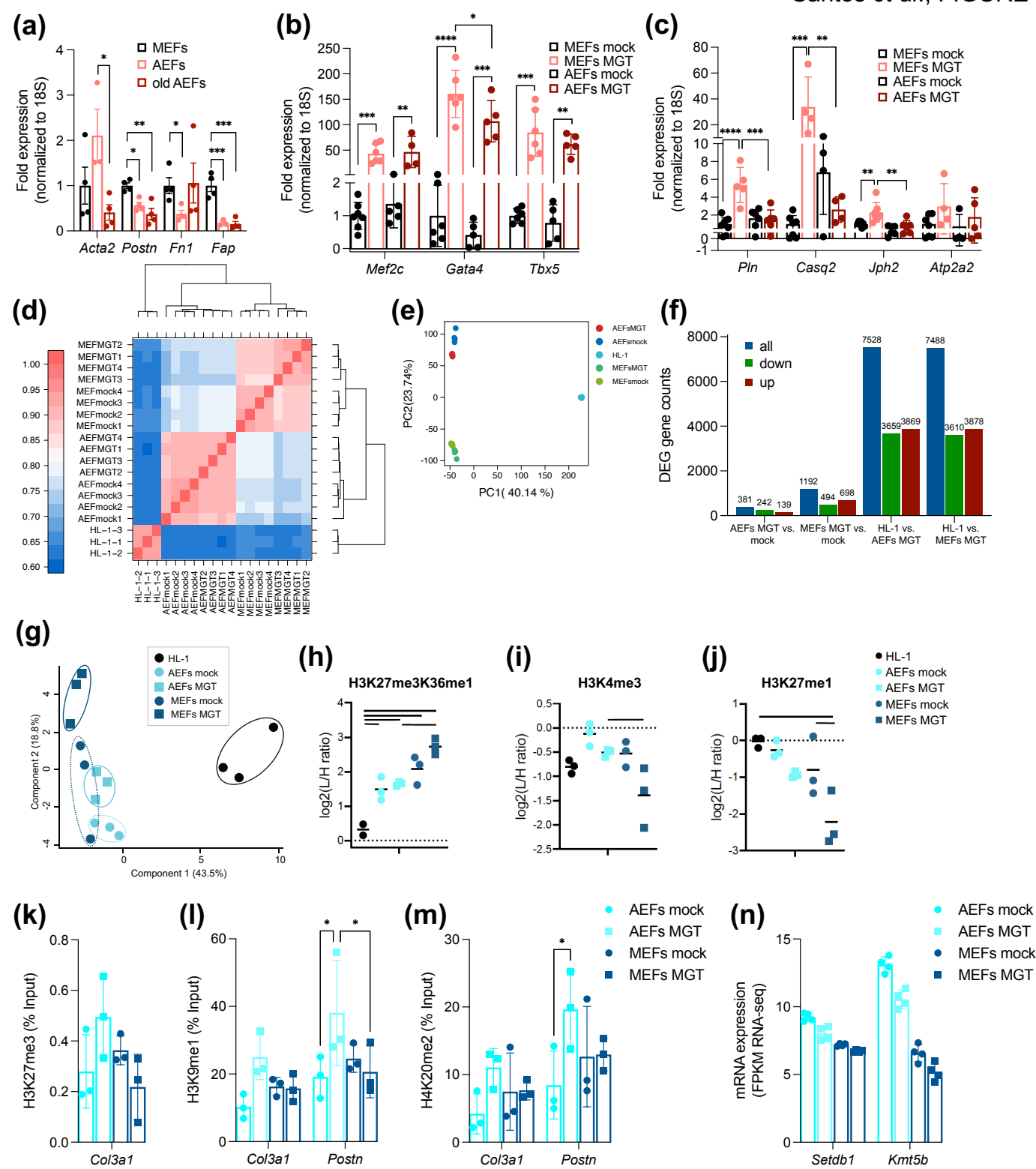

Supplement: Supplementary file 1 — Appendix S1. [file ACEL-24-e14371-s002.zip › FigS1_Santos et al.pdf]

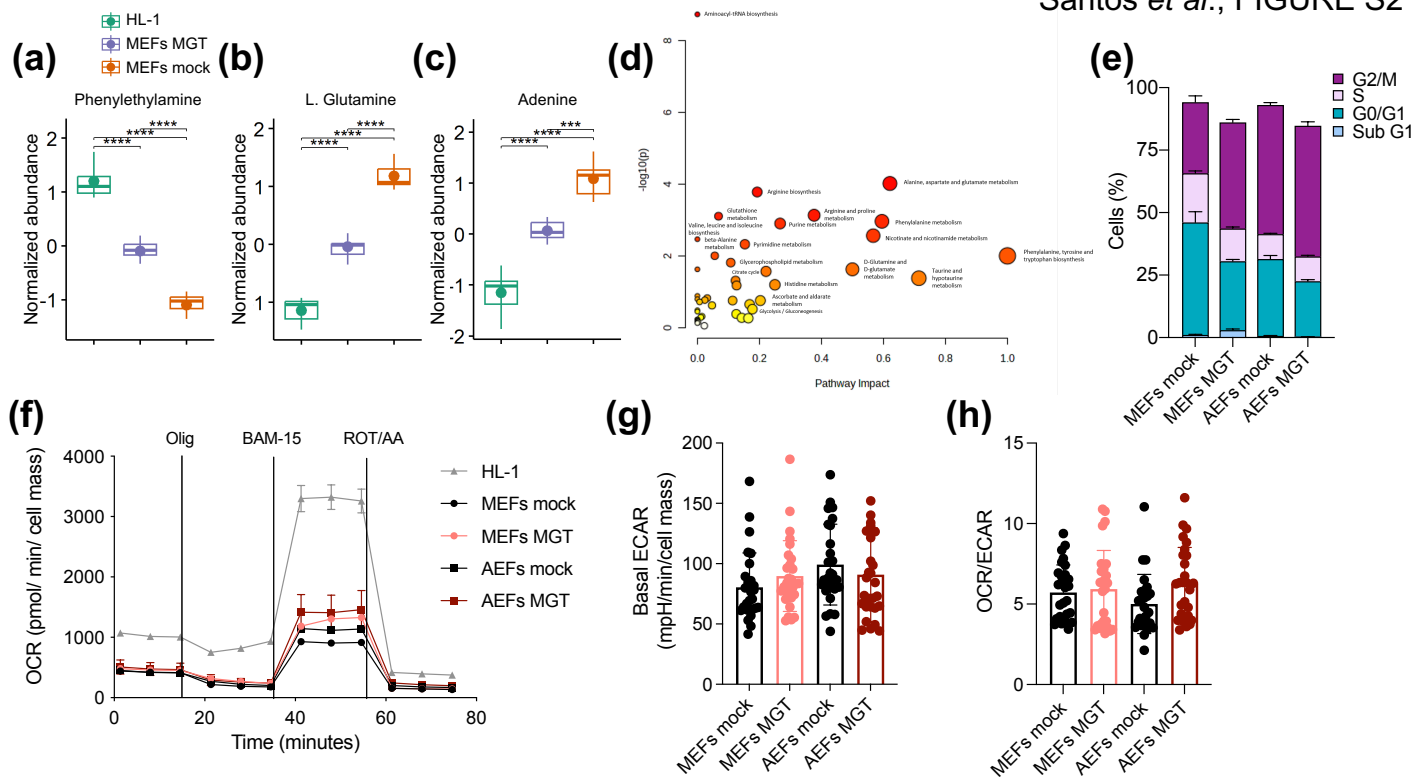

Supplement: Supplementary file 1 — Appendix S1. [file ACEL-24-e14371-s002.zip › FigS2_Santos et al.pdf]

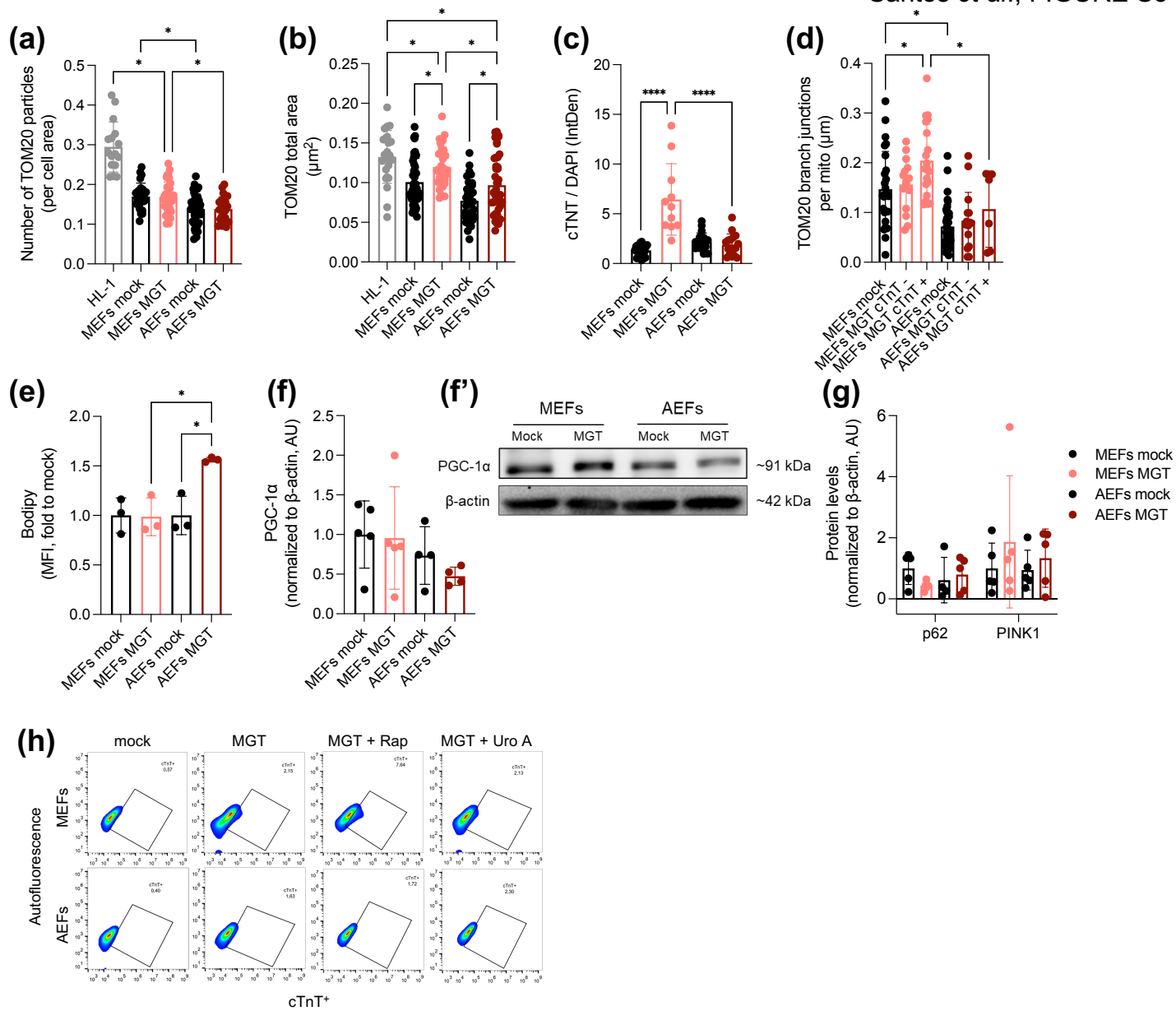

Supplement: Supplementary file 1 — Appendix S1. [file ACEL-24-e14371-s002.zip › FigS3_Santos et al.pdf]

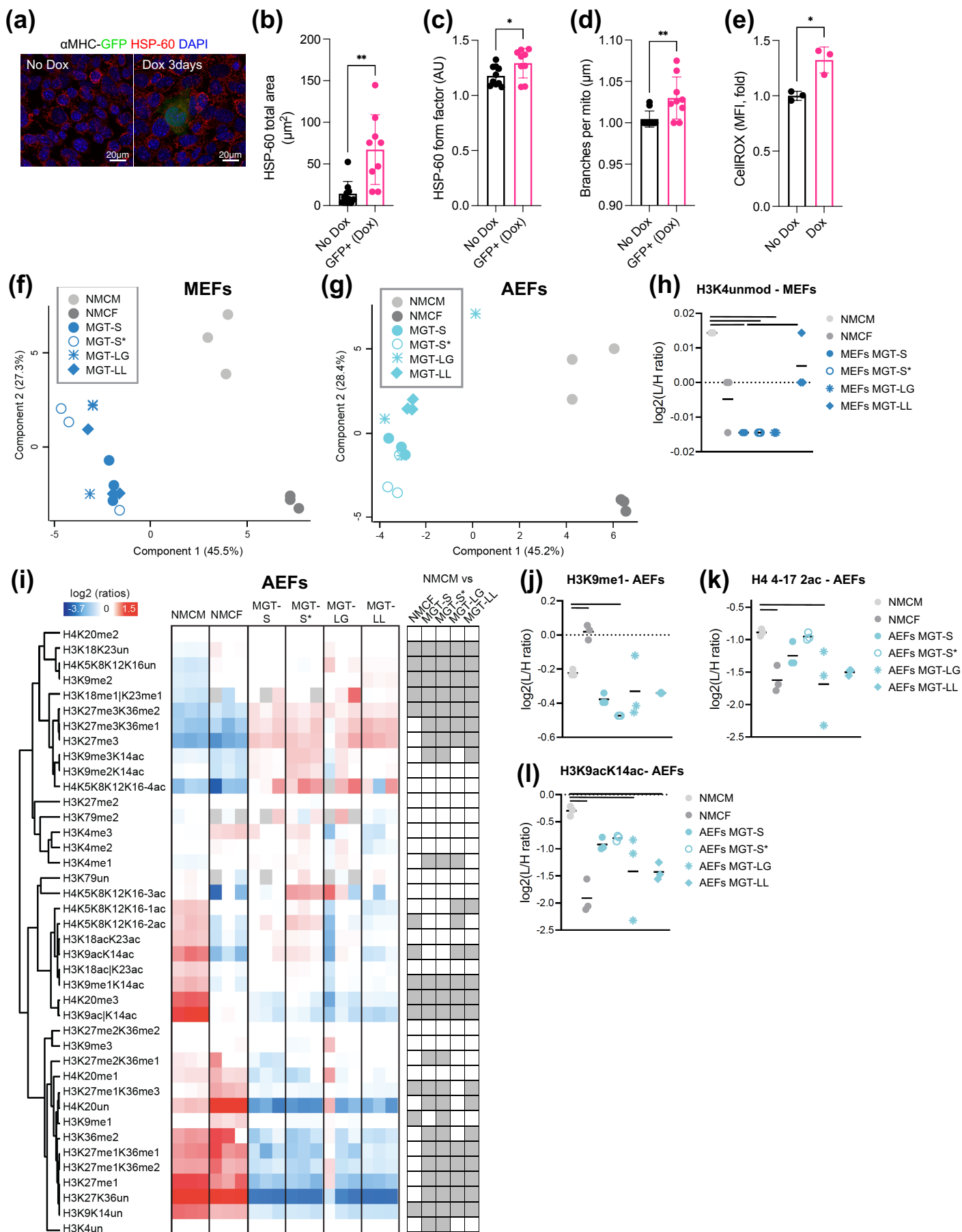

Supplement: Supplementary file 1 — Appendix S1. [file ACEL-24-e14371-s002.zip › FigS4_Santos et al.pdf]

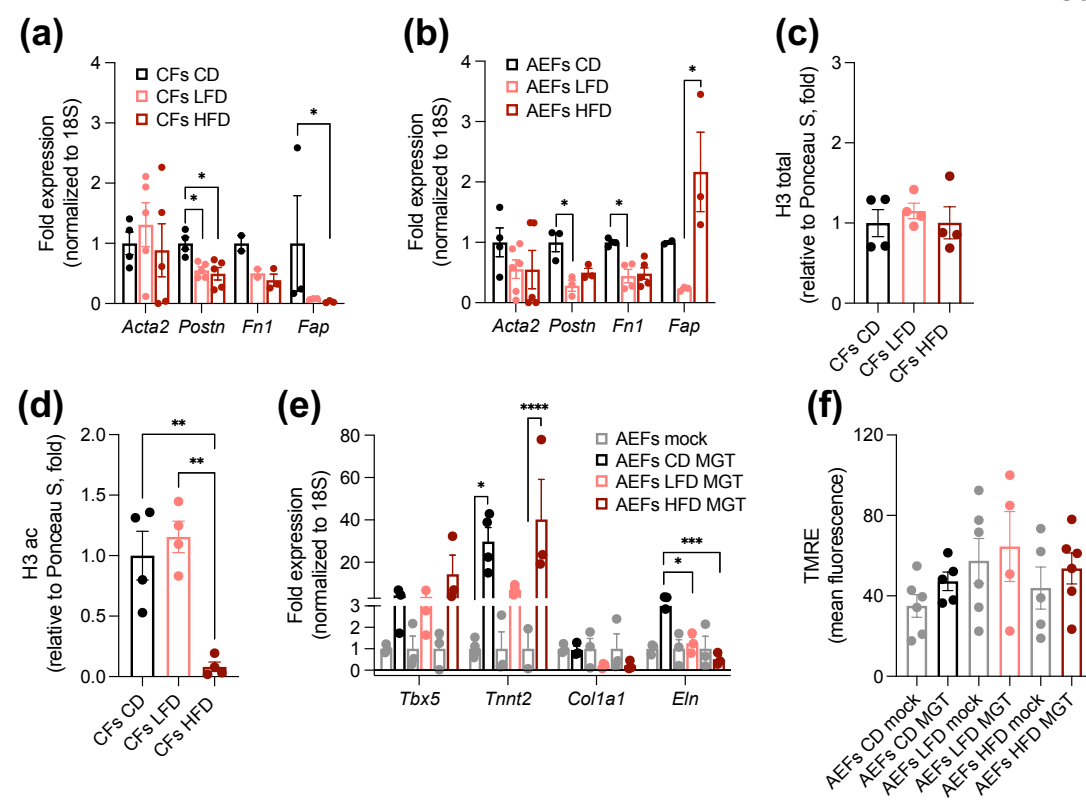

Supplement: Supplementary file 1 — Appendix S1. [file ACEL-24-e14371-s002.zip › FigS5 Santos et al.pdf]
