# Supplementary material for: Age‐associated metabolic and epigenetic barriers during direct reprogramming of mouse fibroblasts into induced cardiomyocytes
Source: Aging Cell. 2024 Nov 14;24(2):e14371. doi: 10.1111/acel.14371 (PMC11822649; doi:10.1111/acel.14371)
Supplement: Supplementary file 3 — Movie S2. [file ACEL-24-e14371-s001.zip › Movie S2.docx]

Movie S2. Spontaneous Ca^2+^ oscillations by Rhod-3 labeling in HL-1 mouse cardiomyocyte cell line.
