# Supplementary material for: Age‐associated metabolic and epigenetic barriers during direct reprogramming of mouse fibroblasts into induced cardiomyocytes
Source: Aging Cell. 2024 Nov 14;24(2):e14371. doi: 10.1111/acel.14371 (PMC11822649; doi:10.1111/acel.14371)
Supplement: Supplementary file 1 — Appendix S1. [file ACEL-24-e14371-s002.zip › Santos et al_in preparation Supp data AC_rebuttal.docx]

**SUPPLEMENTARY EXPERIMENTAL PROCEDURES**

**Isolation of mouse primary fibroblasts and cardiomyocytes**

Primary fibroblasts were isolated as previously reported (Nóbrega-Pereira & de Jesus 2020). Briefly, mouse embryonic fibroblasts (MEFs) were prepared from E13.5 embryos, whereas adult skin dermal ear-derived fibroblasts (AEFs) were isolated from chow-diet fed adult male with 4-6 or 12 months or 18-week-old animals (4.5 months) at the end of the CD, HFD, LFD dietary regimens. Isolation of neonatal (day 1-3) mouse cardiomyocytes (NMCMs) and cardiac fibroblasts (NMCFs) was performed according to (Lu et al. 2022). Briefly, homogenized hearts were transferred to a gentleMACS™ C Tube (Miltenyi Biotec) and washed twice with PBS 1x. The enzyme mix from Neonatal Heart Dissociation Kit (Miltenyi Biotec) was added and placed into the gentleMACS™ Dissociator (Miltenyi Biotec) for 1 h. Growth medium (MEM, Bioconcept) supplemented with 5% FBS (Gibco), 1% penicillin-streptomycin (Life Technologies) was added and the content was pulled through a 70 µm cell strainer and centrifuged at 300 × g for 5 min. For NMCMs, cardiomyocytes present in the supernatant of the pre-plating mix were counted and plated onto 10 cm plates previously coated with 0.02% gelatin porcine (SIGMA) with growth medium at 37ºC with 1% CO_2_. For NMCFs, DMEM was supplemented with 10% FBS (Gibco), added to the adhered cells and incubated at 37°C with 5% CO_2_.

For isolation of adult mouse CFs, mouse hearts were washed with ice-cold HBSS 1x with 1 % penicillin-streptomycin (Gibco), cut and pooled into a 10 cm dish, minced with a scalpel, incubated with digestion buffer [Collagenase II (Worthington)/ Dnase I (SIGMA)/0.05% Trypsin-EDTA in 12 mL of HBSS] for 15 min at 37°C with 5% CO_2_, followed by dissociation by pipetting. The digested solution was passed through a 100 µm cell strainer and centrifuged at 100 × g

for 2 min, followed by supernatant centrifugation at 300 × g for 10 min. The pellet was resuspended in Fibroblast Growth Medium [FGM-3 (Promocell), supplemented with 10% FBS (Gibco), 1% penicillin-streptomycin (Gibco), insulin and FGF (Promocell)], and seeded onto 10 cm plates previously coated with 0.1% gelatin porcine (SIGMA).

**RNA-sequencing**

Total RNA was extracted using GRS Total RNA Kit (GRiSP) according to manufacturer’s instructions. RNA concentration and purity was measured using NanoDrop 2000 (Thermo Fisher Scientific). RNA integrity was assessed using the RNA Nano 6000 Assay Kit of the Agilent Bioanalyzer 2100 system (Agilent Technologies).

For library preparation and transcriptome sequencing, a total amount of 1 μg RNA per sample was used as input material for the RNA sample preparations. Sequencing libraries were generated using NEBNext UltraTM RNA Library Prep Kit for Illumina (NEB) following manufacturer’s recommendations and index codes were added to attribute sequences to each sample. Briefly, mRNA was purified from total RNA using poly-T oligo-attached magnetic beads. Fragmentation was carried out using divalent cations under elevated temperature in NEBNext First Strand Synthesis Reaction Buffer (5X). First strand cDNA was synthesized using random hexamer primer and M-MuLV Reverse Transcriptase. Second strand cDNA synthesis was subsequently performed using DNA Polymerase I and RNase H. Remaining overhangs were converted into blunt ends via exonuclease/polymerase activities. After adenylation of 3’ ends of DNA fragments, NEBNext Adaptor with hairpin loop structure were ligated to prepare for hybridization. In order to select cDNA fragments of preferentially 240 bp in length, the library fragments were purified with AMPure XP system (Beckman Coulter). Then 3 μL USER Enzyme (NEB, USA) was used with size-selected, adaptor-ligated cDNA at 37°C for 15 min followed by 5 min at 95°C before PCR. Then PCR was performed with Phusion High-Fidelity DNA polymerase, Universal PCR primers and Index (X) Primer. At last, PCR products were purified (AMPure XP system) and library quality was assessed on the Agilent Bioanalyzer 2100 system.

The clustering of the index-coded samples was performed on a cBot Cluster Generation System using TruSeq PE Cluster Kit v4-cBot-HS (Illumia) according to the manufacturer’s instructions. After cluster generation, the library preparations were sequenced on an Illumina platform and paired-end reads were generated. Raw data (raw reads) of fastq format were firstly processed through in-house perl scripts. In this step, clean data(clean reads) were obtained by removing reads containing adapter, reads containing ploy-N and low quality reads from raw data. At the same time, Q20, Q30, GC-content and sequence duplication level of the clean data were calculated. All the downstream analyses were based on clean data with high quality. For comparative analysis, the adaptor sequences and low-quality sequence reads were removed from the data sets. Raw sequences were transformed into clean reads after data processing. These clean reads were then mapped to the reference genome sequence. Only reads with a perfect match or one mismatch were further analyzed and annotated based on the reference genome. Spearman's correlation coefficient R (Spearman's Correlation Coefficient) was applied to evaluate reproducibility of biological replicates. Hisat2 tools soft were used to map with reference genome. Quantification of gene expression levels were estimated by fragments per kilobase of transcript per million fragments mapped (FPKM). The formula is shown as follow:  FPKM=cDNA Fragments / Mapped Fragments (Millions) x Transcript Length(kb). PCA was performed on FPKM of each sample. Differential expression analysis of two conditions/groups with biological replicates was performed using the DESeq2 (Subramanian et al. 2005). The resulting p values were adjusted using the Benjamini and Hochberg’s approach for controlling the false discovery rate (FDR). Genes with an adjusted p-value < 0.01 found by DESeq2 were assigned as differentially expressed. Similarity among samples were displayed by reducing dimensionality into two or three principal components. Gene Ontology (GO) enrichment analysis of the differentially expressed genes (DEGs) was implemented by the GOseq R packages based Wallenius non-central hyper-geometric distribution (Young et al. 2010) which can adjust for gene length bias in DEGs. Data has been deposited to the European Nucleotide Archive with the dataset identifier Project PRJEB77697 and Study ERP162057.

**Western blotting**

Whole-cell extracts were prepared using RIPA buffer (Thermo Fisher Scientific) containing protease and phosphatase inhibitors (Thermo Fisher Scientific) by centrifugation at 15,100  × g for 10 min at 4°C. Histone-enriched (acidic) proteins extracts were prepared using Histone Extraction Kit (Abcam ab113476) following manufacturer instructions. Protein concentration was measured using Pierce BCA Protein Assay Kit (Thermo Fisher Scientific) and a plate-reader Tecan Infinite M200 (Tecan). Samples were prepared by boiling with NuPAGE LDS sample buffer (Invitrogen) for 5 min at 95°C in the presence of 50 mM DTT and protein standard (NZYTech, MB09002). 5-15 μg of total or histone-enriched protein was subjected to SDS-PAGE in homemade 10% or 17% Tris-glycine SDS-PAGE gels. Proteins were wet-transferred onto a nitrocellulose membrane (Amersham) in Tris-glycine and methanol (20% v/v), stained with Ponceau S (0.1% (w/v), Carl Roth) and subsequently blocked with 5% BSA (prepared in TBS-tween20 0.05%), for 1 h at RT. Membranes were incubated overnight at 4°C with mouse anti-PGC-1α (Calbiochem, KP9803, 1:1000), mouse anti-PINK1 (Santa Cruz Biotechnology, sc-517353, 1:500), rabbit anti-p62 (Cell Signaling 5114, 1:1000), rabbit anti-histone H3 (Abcam ab1791, 1:2000), rabbit anti-pan histone H3 (acetyl K9 + K14 + K18 + K23 + K27) (Abcam ab47915, 1:1000), followed by incubation with either anti-mouse or anti-rabbit HRP-conjugated secondary antibodies (Invitrogen, G21040 and G21234, respectively, 1:10000) or HRP-conjugated rabbit anti-β-actin (Invitrogen MAB-32540, 1:1000). The Pierce ECL (Amersham, RPN2235) detection system and Chemidoc (Bio-Rad) was used for detection. Quantification of densitometric units was performed using Image Lab Ver. 6.1 software (RRDI:SCR_014210) and uncropped membranes are presented at Supplementary Figure S6.

**Seahorse XFe96 Extracellular Flux Analysis**

Cells were dissociated, counted and plated in the Seahorse XF96 Cell Culture Microplates (Bioscience), coated with 0.1% gelatin porcine (SIGMA) at a density of 1 x 10^4^ cells/well. On the next day, the Seahorse Cell Mito Stress Test profile was performed using the Seahorse XFe96 Extracellular Flux Analyzer (Agilent Scientific Instruments) (Amorim et al. 2022). Briefly, cell culture medium was replaced by pre-warmed low-buffered serum-free minimal DMEM (D5030, SIGMA) medium supplemented with 1 mM pyruvate, 2 mM glutamine, and 10 mM glucose (pH 7.4), and cells were incubated in a non-CO_2_ incubator at 37ºC for 1 h. Oxygen consumption rate (OCR) was measured under standard conditions and after the sequential addition of oligomycin (2 μM) (port A), BAM-15 (2 μM) (port B) and rotenone (1 μM) plus antimycin A (1 μM) (port C). Three baseline rate measurements of cell OCR were made using a 3 min mix, 3 min measure cycle. The compounds were then pneumatically injected by the XFe96 Analyzer into each well, mixed and OCR measurements made using a 3 min mix, 3 min measure cycle. Results were normalized by cell mass density through the sulforhodamine B (SRB) assay for cell mass determination. Briefly, cells were fixed with 10% (w/v) TCA overnight at 4ºC, plates were dried in an oven at 37ºC followed by addition of 0.05% SRB in 1% acetic acid solution and incubation at 37°C for 1 h. The wells were washed with 1% acetic acid in water and dried, followed by addition of Tris (pH 10) and optical density measurement at 540 nm in Biotek Cytation 3 reader (Biotek Instruments) and analyzed using the Wave Desktop 2.6.1 software (RRID:SCR_014526).

**Metabolite extraction and Liquid Chromatography-Mass Spectrometry (LC–MS) analysis**

About 4 x 10^6^ cells were used to extract metabolites and soluble lipids, with 80% (v/v) methanol (at -80°C or on dry ice or liquid nitrogen) as previously described (Yuan et al. 2012). Pellets were kept at -80ºC until extraction. The dried samples were resuspended in 50 μL of LC/MS phase A containing an internal standard (Leu-Tyr, 0.02 mg/mL, SIGMA). Quality control (QC) samples, created by pooling aliquots of all samples, were produced. 5 μL of each sample were analyzed by C18-LC-MS using an Ascentis® Express 90 Å C18 HPLC column (15 cm x 2.1 mm; 2.7 μm, Supelco®) with an HPLC system (Ultimate 3000 Dionex, Thermo Fisher Scientific) coupled online to a Q-Exactive™ Mass Spectrometer (Thermo Fisher Scientific). The solvent system consisted of two mobile phases, a mobile phase A (0.1% formic acid, 2% acetonitrile in water) and mobile phase B (B: 0.1% formic acid, 100% acetonitrile. Metabolites were eluted with a 100% of mobile phase A for 2 min, followed by a linear gradient of 0–100% B over 18 min and held for 2 min at a flow rate of 260 mL/min and a temperature of 35ºC. The mass spectrometer (MS) was operated as previously described (Maurício et al. 2022). The Xcalibur data system (v3.3, Thermo Fisher Scientific) was utilized for data acquisition. Metabolites were identified using MS-DIAL v 4.70 software (Mass Spectrometry-Data Independent Analysis (MS-DIAL) (Tsugawa et al. 2020). The identification of metabolites was conducted in both negative and positive ionization modes, utilizing the QC raw files obtained from the MS/MS analysis for each mode. Validated species were integrated and quantified using MZmine v 2.53 software (Pluskal et al. 2010). Univariate and multivariate statistical analyses were carried out using R version 4.2.3 in Rstudio version 2022.12.0. Principal component analysis (PCA) was conducted using the R libraries FactoMineR (Lê et al. 2008) and factoextra.

**Histone extraction and LC–MS-based analysis of histone acetylation and methylation marks**

Histone were enriched from 1-2 x 10^6^ cells using the Histone extraction kit (Abcam) according to the manufacturer’s instructions. Protein extracts were resuspended in Balanced-DTT Buffer and stored at -80ºC until analysis. Histone protein concentration and purity was accessed by BCA protein quantification assay (Thermo Fisher Scientific) and SDS-PAGE analysis with 17% polyacrylamide gel and Coomassie staining (0.12% (w/v) Coomassie G-250 in 20% methanol (v/v)) using known amounts of histone H3.1 recombinant protein (EpiCypher, Inc). Approximately 4 µg of histone octamer were mixed with an equal amount of heavy-isotope labeled histones, which were used as an internal standard, (Noberini & Bonaldi 2017) and separated on a 17% SDS-PAGE gel. Histone bands were excised, chemically acylated with propionic anhydride and in-gel digested with trypsin, followed by peptide N-terminal derivatization with phenyl isocyanate (PIC) (Noberini et al. 2021). Peptide mixtures were separated by reversed-phase chromatography on an EASY-Spray column (Thermo Fisher Scientific), 25-cm long (inner diameter 75 µm, PepMap C18, 2 µm particles), which was connected online to a Q Exactive Plus instrument (Thermo Fisher Scientific) through an EASY-Spray™ Ion Source (Thermo Fisher Scientific). The acquired RAW data were analyzed using EpiProfile 2.0 (Yuan et al. 2018) followed by manual validation. For each histone modified peptide, a % relative abundance (%RA) value for the sample (light channel - L) or the internal standard (heavy channel - H) was estimated by dividing the area under the curve of each acetylated peptide for the sum of the areas corresponding to all the observed forms of that peptide and multiplying by 100 and Light/Heavy (L/H) ratios of %RA were then calculated. The mass spectrometry data has been deposited to the ProteomeXchange Consortium (Vizcaíno et al. 2014) via the PRIDE partner repository with the dataset identifier PXD046542.

**Microscopy imaging**

The images and videos were obtained through a 63x oil objective of the Zeiss LSM 880 confocal fluorescence microscope and analyzed with ImageJ software (RRID:SCR_003070). After determining cell boundary, the fluorescence intensities were measured as integrated density (IntDen). Colocalization analyses were performed on fields of view using the Pearson’s coefficient on the JACoP plug-in. Mitochondrial network and morphology was evaluated with Mitochondrial Analyzer plug-in (Chaudhry et al. 2020). Briefly, a z-stack of images (with an interval of 1,26 μm) was acquired and maximum intensity projections were obtained from each z-stack. For each experiment, the same threshold was applied across groups and analysis was performed blinded. The corrected total cell fluorescence (CTCF) was analyzed in ImageJ (cell counter plugin) and calculated according to: CTCF = Integrated Density – (Area of selected cell x Mean fluorescence of background readings. TMRE mean fluorescence intensity levels were analyzed in the Mitometer software under optimized conditions (Lefebvre et al. 2021). The Ca^2+^ flashing cells were determine qualitatively (presence/absence of transient calcium signals) by a blinded operator in 3 independent wells from 1 representative experiment and videos of calcium oscillation were recorded with 194 frames imaged (experiment duration = 63 seconds) in an area of 67.5x 67.5 µm, with a resolution of 7.5879 pixels per µm. Rhod-3 intensity traces are representative of average cellular intensity variation during experiment, with background subtracted to normalize for focal drift and photobleaching.

**SUPPLEMENTARY FIGURE LEGENDS AND TABLES**

FIGURE S1. Transdifferentiation of mouse fibroblasts into iCMs is accompanied by epigenetic transitions. (a-c) qPCR analysis of the relative expression of the indicated genes in parental MEFs, AEFs and old AEFs (a), mock or MGT retroviral-transduced at D11/12 (b, c) (fold to MEFs mock). (d-f) Bulk RNA-sequencing analysis in mock and MGT-transduced MEFs and AEFs at D11, and HL-1 mouse cardiomyocyte cell line. Correlation heatmap (d) and Principal component analysis (PCA) with a scatterplot of PC1 versus PC2 (e) of the indicated samples. Differentially expressed genes (DEGs) displaying number for all, down- and up- regulated between the display set of conditions (f). (g-j) Mass spectrometry-based histone acetylation and methylation proteomic bulk analysis in mock and MGT-transduced MEFs and AEFs at D11, and HL-1 mouse cardiomyocyte cell line. Principal component analysis (PCA) based on histone PTM data obtained from the z scores of the samples shown in Figure 2a (g). Selected histone modified peptides showing the levels of tri-methylated form of H3 lysine 27 and mono-methylated lysine 36 (h), tri-methylated form of H3 lysine 4 (i) and mono-methylated form of H3 lysine 27 (j), the bar indicates a p-value < 0.05. (k-m) ChIP-qPCR for H3K27me3 (k), H3K9me1 (l) and H4K20me2 (m) for fibroblast genes (*Col3a1*, *Postn*) on mock and MGT-transduced MEFs and AEFs on D11 expressed as % to Input and comparisons to corresponding mock for each gene. (n) Bulk RNA-sequencing difference analysis (without log2fc and FDR filtering) for the expression amount of *Kmt5b* and *Setdb1* in mock and MGT-transduced MEFs and AEFs at D11. Each point on the plot indicates individual measurements and mean ± s.d of n = 3-7 biological replicates from one representative experiment or three independent experiments. p values were calculated by one-way ANOVA with False Discovery Rate followed by Tukey’s post-test for multiple comparisons. *p < 0.05; **p < 0.01; ***p < 0.001; ****p < 0.0001. Data analysis and visualization were performed using GraphPad Prism (RRID:SCR_002798), Perseus (RRID:SCR_015753) (g-j), R project for statistical computing (RRID:SCR_001905) prcomp (d) and pheatmap (e).

FIGURE S2. Transdifferentiation of mouse fibroblasts into iCMs is accompanied by metabolic and bioenergetic transitions resembling those present in cardiomyocytes. (a-d) LC-MS-based untargeted metabolomics bulk analysis of MEFs mock or MGT-transduced at D11, and the HL-1 mouse cardiomyocyte cell line. Selected box-plot analysis relative abundances of the identified metabolites phenylethylamine (a), L-glutamine (b) and adenine (c) between HL-1 (green boxplots), MEFs mock (orange boxplots) and MEFs MGT (purple boxplots). Dot plot of identified metabolomic pathways in MEFs mock and MGT displaying log(p) values from pathway enrichment analysis vs. values of the pathways impact, where higher impact values represent the relative importance of the pathway, the size of the circle indicates the impact of the pathway while the color represents the significance (d). (e) Cell cycle analysis of propidium iodide staining by flow cytometry displaying the percentage of cells in each phase (sub-G1, G0/G1, S and G2/M) in MEFs and AEFs mock or MGT transduced at D11. (f-h) Oxygen consumption rate (OCR) measurement in MEFs and AEFs mock or MGT transduced or HL-1 at D11 using Seahorse XF96 Cell Mito Stress Test (f), basal extracellular acidification rate (ECAR) (g) and OCR/ECAR ratio (h). Data were normalized to cell mass using the sulforhodamine B (SRB) assay. Each point on the plot indicates individual measurements and mean ± s.d of n = 3-7 biological replicates from one representative experiment (a-e) or n=26-28 technical replicates from three independent experiments (f-h). p values were calculated by one-way ANOVA and Kruskal-Wallis tests with False Discovery Rate multiple comparisons test by post-hoc Tukey’s or Dunn's test. ***p < 0.001; ****p < 0.0001. Data analysis and visualization were performed using GraphPad Prism (RRID:SCR_002798), R package ggplot2 (RRID:SCR_014601) (a-c) and MetaboAnalyst 5.0 (RRID:SCR_015539) (d).

FIGURE S3. Extensive remodeling of mitochondrial network and mitophagy takes place during direct cardiac conversion. (a-b) Analysis of the number of TOM20 particles (a) and total area (b) in MEFs and AEFs mock or MGT-transduced at D11 or HL-1 cells. (c) Quantification of immunofluorescence staining for cTNT per cell (integrated density, IntDen) in MEFs and AEFs mock or MGT-transduced at D11. (d) Analysis of TOM20 branch junctions per mitochondria in MGT-transduced cTNT positive and negative cells (20 cTNT^+^/36 total cells for MEFs MGT and 6 cTNT^+^/22 total cells for AEFs MGT) or mock cells at D11. (e) Flow cytometry quantification of BODIPY 493/503 depicted as relative median fluorescence intensity (MFI, fold to corresponding mock) in MEFs and AEFs mock or MGT-transduced at D11. (f-f’) Immunoblotting analysis for PGC-1α and β-actin in whole cell extracts depicting densitometric quantification (f, normalized to β-actin) and representative images (f’, uncropped blots in Figure S6) MEFs and AEFs mock or MGT-transduced at D11. (g) Densitometric quantification of immunoblotting analysis for p62 and PINK1 normalized to β-actin (related to Figure 4j) in MEFs and AEFs mock or MGT-transduced at D11. (h) Representative flow cytometry plots of cardiac troponin (cTnT) in MEFs and AEFs mock or MGT retroviral-infected on D11, untreated or supplemented with rapamycin (Rap, 10 nM) or urolithin A (UroA, 5 µM) (related to Figure 4k).

Each point on the plot indicates individual measurements and mean ± s.d of n = 6-43 (a-d) cells analyzed or n=3-5 biological replicates from one representative or two independent experiments. p values were calculated by Kruskal Wallis with false discovery rate correction and one-way ANOVA and Tukey’s post-test for multiple comparisons. *p < 0.05 between the indicated groups. Graphs were created using GraphPad Prism (RRID:SCR_002798) and flow cytometry plots (h) with FlowJo (RRID:SCR_008520).

FIGURE S4. Metabolic modulation can bypass epigenetic and age-associated barriers to DCC. (a) Representative images of α-MHC-GFP, mitochondria HSP-60 and DAPI in icMEFs with no Dox or Dox for 3 days. (b-d) Analysis of HSP-60 fluorescence corrected by cell area (b), form factor (shape, c) and branches per mitochondria (network connectivity, d) in icMEFs with no Dox or Dox (GFP-expressing cells) for 3 days. (e) Flow cytometry quantification of CellROX (ROS) depicted as the fold change of the relative median fluorescence intensity (MFI) in icMEFs with no Dox or Dox for 3 days and in the presence of Resveratrol (RVT, 20 nM). (f-l) Mass spectrometry-based histone acetylation and methylation proteomic bulk analysis in neonatal mouse cardiac myocytes (NMCM) and fibroblasts (NMCF) and MGT-transduced MEFs and AEFs at D11 in the presence of standard medium (S: glucose 25 mM, FBS 10%) only or supplemented with sodium acetate and α-KG (S*: sodium acetate 5 mM and α-KG 1,5 mM), low glucose (LG: glucose 1 mM, FBS 10%) or low lipids (LL: glucose 25 mM, FBS 1%) growth medium. PCA based on histone PTM data obtained from the z scores of the samples shown in the heatmap display of histone PTM levels for MGT-transduced MEFs (f) and AEFs (g). Levels of unmodified form of H3 lysine 4 in the indicated samples, the bar indicates a p-value < 0.05 (h). Heatmap display of histone PTM levels in AEFs samples, NMCM and NMCF clustered based on Pearson’s correlation, grey squares indicates peptides that were not quantified. The panel on the right shows significant changes (grey) between NMCM and the indicated comparisons (i). Display of selected histone modified peptides showing the levels of mono-methylated form of H3 lysine 9 (j), bi-acetylated form of H4 lysine 4–17 (k) and mono-methylated form of H3 lysine 9 and mono-acetylated lysine 14 (l) in the indicated samples, the bar indicates a p-value < 0.05. Each point on the plot indicates individual measurements and mean ± s.d of n = 9-10 cells analyzed (b-d) or n = 3-5 biological replicates from two independent or one representative experiment. p values were calculated by unpaired t test or one-way ANOVA or Kruskal Wallis with False Discovery Rate correction by post-hoc Tukey’s test. *p < 0.05; **p < 0.01 . Graphs were created using GraphPad Prism (RRID:SCR_002798) and Perseus (RRID:SCR_015753) (f-l). Scale bar, 20 µm in (a).

FIGURE S5. Dietary lipids improve DCC of adult fibroblasts *ex vivo*. (a-b) qPCR analysis of the relative expression of the indicated genes in CFs (a) and AEFs (b) isolated from animals under CD, HFD and LFD. (c-d) Densitometric quantification of immunoblotting analysis for total histone H3 (c) and H3 pan-acetylated (acetyl K9 + K14 + K18 + K23 + K27) (d) normalized to Ponceau S staining (fold), from whole cell extracts of CFs isolated from animals under CD, HFD and LFD. (e) qPCR analysis of the relative expression of the indicated genes in AEFs isolated from animals under CD, HFD and LFD subjected to mock or MGT transduction at D11/12 (fold to corresponding mock). (f) TMRE quantification depicted as relative mean fluorescence intensity in AEFs isolated from animals under CD, HFD and LFD subjected to mock or MGT transduction at D11. Each point on the plot indicates individual measurements and mean ± s.e.m of n = 3-9 biological replicates from one representative or two independent experiments. p values were calculated by one-way ANOVA with False Discovery Rate followed by Tukey’s post-test for multiple comparisons or Kruskal-Wallis test. *p < 0.05; **p < 0.01; ***p < 0.001; ****p < 0.0001. Graphs were created using GraphPad Prism (RRID:SCR_002798).

FIGURE S6. Uncropped membranes relative to the immunoblotting for PGC-1α, p62, PINK1 and β-actin displayed in Figure S3f’ and Figure 4j and total Histone H3, H3 pan-acetylated (acetyl K9 + K14 + K18 + K23 + K27) or Ponceau S displayed in Figure 6e’.

TABLE S1**.** Bulk RNA-sequencing analysis in mock and MGT-transduced MEFs and AEFs at D11 with selected GO terms of biological processes enriched (up) or suppressed (down) in MEFs MGT versus mock or AEFs MGT versus mock.

TABLE S2. Mass spectrometry-based histone acetylation and methylation proteomic bulk analysis in mock and MGT-transduced MEFs and AEFs at D11, and HL-1 mouse cardiomyocyte cell line. Light/Heavy (L/H) ratios of % relative abundance (%RA) value for the indicated samples.

TABLE S3. LC-MS-based untargeted metabolomics of MEFs mock- (control) or MGT-transduced at D11, and the HL-1 mouse cardiomyocyte cell line. List of 99 validated metabolic species identified and relative abundance.

TABLE S5. Mass spectrometry-based histone acetylation and methylation proteomic bulk analysis in neonatal mouse cardiac myocytes (NMCM) and fibroblasts (NMCF), MEFs and AEFs MGT retroviral-infected at D11 in the presence of standard (S: glucose 25 mM, FBS 10%), supplemented with sodium acetate and α-KG (S*: sodium acetate 5 mM and α-KG 1,5 mM), low glucose (LG: glucose 1 mM, FBS 10%) or low lipids (LL: glucose 25 mM, FBS 1%) growth media formulation. Light/Heavy (L/H) ratios of % relative abundance (%RA) value for the indicated samples.

MOVIE S1. Spontaneous Ca^2+^ oscillations by Rhod-3 labeling in MGT-transduced MEFs at 4 weeks post-transduction (D25).

MOVIE S2. Spontaneous Ca^2+^ oscillations by Rhod-3 labeling in HL-1 mouse cardiomyocyte cell line

MOVIE S3. Spontaneous Ca^2+^ oscillations by Rhod-3 labeling in MGT-transduced AEFs at 4 weeks post-transduction (D25).

TABLE S4. LC-MS-based untargeted metabolomics of MEFs mock- (control) or MGT-transduced at D11, and the HL-1 mouse cardiomyocyte cell line. Differential analysis of cellular metabolites was associated with top 18 significantly altered metabolic pathways, in terms of -log(p value) by FDR.

| **Pathway** | **p-Value** | **Metabolites** | **FDR** | **-Log(p)** | **Impact** |
| --- | --- | --- | --- | --- | --- |
| Aminoacyl-tRNA biosynthesis | 1.8613e-9 | 14/48 | 1.5635e-7 | 8.7302 | 0.0 |
| Alanine, aspartate and glutamate metabolism | 9.4806e-5 | 7/28 | 0.0039819 | 4.0232 | 0.621 |
| Arginine biosynthesis | 1.6352e-4 | 5/14 | 0.0045785 | 3.7864 | 0.19289 |
| Arginine and proline metabolism | 7.2478e-4 | 7/38 | 0.012954 | 3.1398 | 0.37647 |
| Glutathione metabolism | 7.7104e-4 | 6/28 | 0.012954 | 3.1129 | 0.06811 |
| Phenylalanine metabolism | 0.0010706 | 4/12 | 0.014767 | 2.9704 | 0.59524 |
| Purine metabolism | 0.0012306 | 9/66 | 0.014767 | 2.9099 | 0.26679 |
| Nicotinate and nicotinamide metabolism | 0.0026847 | 4/15 | 0.028189 | 2.5711 | 0.56711 |
| Valine, leucine and isoleucine biosynthesis | 0.0033639 | 3/8 | 0.031396 | 2.4732 | 0.0 |
| Pyrimidine metabolism | 0.0046484 | 6/39 | 0.039047 | 2.3327 | 0.15316 |
| beta-Alanine metabolism | 0.0097417 | 4/21 | 0.068525 | 2.0114 | 0.05597 |
| Phenylalanine, tyrosine and tryptophan biosynthesis | 0.0097892 | 2/4 | 0.068525 | 2.0093 | 1.0 |
| Glycerophospholipid metabolism | 0.015089 | 5/36 | 0.097499 | 1.8213 | 0.10756 |
| Nitrogen metabolism | 0.023177 | 2/6 | 0.12979 | 1.6349 | 0.0 |
| D-Glutamine and D-glutamate metabolism | 0.023177 | 2/6 | 0.12979 | 1.6349 | 0.5 |
| Histidine Metabolism | 0.026466 | 3/16 | 0.13895 | 1.5773 | 0.22131 |
| Taurine and hypotaurine metabolism | 0.040987 | 2/8 | 0.20252 | 1.3874 | 0.71428 |
| TCA cycle | 0.047852 | 3/20 | 0.22331 | 1.3201 | 0.12311 |

TABLE S6. List of primers used for RT-qPCR and ChIP-qPCR.

| Name | Sequence |
| --- | --- |
| m*18*S-F | 5’- AGGATGTGAAGGATGGGAAGT-3’ |
| m*18S-*R | 5’- CAGGTCCTCACGCAGCTTG-3’ |
| m*Mef2c*-F | 5’- GAGCCGGACAAACTCAGACA-3’ |
| m*Mef2c*-R | 5’- TCAAAGCTGGGAGGTGGAAC-3’ |
| m*Tnnt2*-F | 5’- GAGCTACAGACTCTGATCGAGG-3’ |
| m*Tnnt2*-R | 5’- CCGCTCATTGCGAATACGC-3’ |
| m*Col3a1*-F | 5’- CTGTAACATGGAAACTGGGGAAA-3’ |
| m*Col3a1*-R | 5’- CCATAGCTGAACTGAAAACCACC-3’ |
| m*Actc1-*F | 5’- GGATTCTGGCGATGGTGTAAC-3’ |
| m*Actc1-*R | 5’-GACCAGCTAGATCCAGGCG-3’ |
| m*Myl7-*F | 5’-TCGGGAGGGTAAGTGTTCC-3’ |
| m*Myl7-*R | 5’-GTCCGTCCCATTGAGCTTCTC-3’ |
| m*Tbx5-*F | 5’-CTTCTATCGCTCGGGCTACC-3’ |
| m*Tbx5-*R | 5’-GCTATAGGAGGGCATGCTGG-3’ |
| m*Col1a1*-F | 5’-GCTCCTCTTAGGGGCCACT-3’ |
| m*Col1a1*-R | 5’-CCACGTCTCACCATTGGGG-3’ |
| m*Eln*-F | 5’-TGTCCCACTGGGTTATCCCAT-3’ |
| m*Eln*-R | 5’-CAGCTACTCCATAGGGCAATTTC-3’ |
| *mActa2*-F | 5’-GTGAAGAGGAAGACAGCACAG-3’ |
| *mActa2*-R | 5’-GCCCATTCCAACCATTACTCC-3’ |
| *mPostn*-F | 5’-CCATTGGAGGCAAACAACTCC-3’ |
| *mPostn*-R | 5’-TTGCTTCCTCTCACCATGCA-3’ |
| *mFn1*-F | 5’-CGTCATTGCCCTGAAGAACA-3’ |
| *mFn1*-R | 5’-AAGGGTAACCAGTTGGGGAA-3’ |
| *mFap*-F | 5’-GTCACCTGATCGGCAATTTGT-3’ |
| *mFap*-R | 5’-TCGTAGATGTAGTATGTCGCTGT-3’ |
| m*Gata4*-F | 5’-TGTGCCAACTGCCAGACTAC -3’ |
| m*Gata4*-R | 5’-TGGGCTTCCGTTTTCTGGTT-3’ |
| m*Pln1*-F | 5’-AAAGTGCAATACCTCACTCGC-3’ |
| m*Pln1*-R | 5’-GGCATTTCAATAGTGGAGGCTC-3’ |
| m*Casq2*-F | 5’-TGCTCATGGTGGGGGTTTATC-3’ |
| m*Casq2*-R | 5’-AGGTTCGTGGTAATAGAGACAGA-3’ |
| m*Jph2*-F | 5’-CCAAGGGCCAGGGTGAATAC-3’ |
| m*Jph2*-R | 5’-TGGCTCCAATATCCCTCAAAGG-3’ |
| m*Atp2a2*-F | 5’-GAGAACGCTCACACAAAGACC-3’ |
| m*Atp2a2*-R | 5’-ACTGCTCAATCACAAGTTCCAG-3’ |
| m*Myh6*-F | 5’-GCCCAGTACCTCCGAAAGTC-3’ |
| m*Myh6*-R | 5’-ATCAGGCACGAAGCACTCC-3’ |
| *mTnnt2* ChIP-F | 5’-GTGTGCAGTCCCTGTTCAGA-3’ |
| *mTnnt2* ChIP-R | 5’-AGGGTGGACACAAGCAAAAG-3’ |
| *mMyh6* ChIP-F | 5’-CACCGTAGCCTATGGCTCTC-3’ |
| *mMyh6* ChIP-R | 5’-CGGCACTCTTAGCAAACCTC-3’ |
| *mPln* ChIP-F | 5’-TACTGCAGCTGCTCCCATAA-3’ |
| *mPln* ChIP-R | 5’-CACGTCTTACCTTGGCTTCG-3’ |
| *mCol3a1* ChIP-F | 5’-TTTGTGCAAAGTGGAACCTG-3’ |
| *mCol3a1* ChIP-R | 5’-TGGACTGCTGTGCCAAAATA-3’ |
| *mPostn* ChIP-F | 5’-TATGCTCTGCTGCTGCTGTT-3’ |
| *mPostn* ChIP-R | 5’-AACAAGCCAGGGACTTACCC-3’ |

**SUPPLEMENTARY REFERENCES**

Amorim R, Cagide F, Tavares LC, Simões RF, Soares P, Benfeito S, Baldeiras I, Jones JG, Borges F, Oliveira PJ & Teixeira J (2022) Mitochondriotropic antioxidant based on caffeic acid AntiOxCIN(4) activates Nrf2-dependent antioxidant defenses and quality control mechanisms to antagonize oxidative stress-induced cell damage. *Free Radic. Biol. Med.* 179, 119–132.

Lê S, Josse J & Husson F (2008) FactoMineR: An R Package for Multivariate Analysis. *J. Stat. Softw.* 25, 1–18. Available at: https://www.jstatsoft.org/index.php/jss/article/view/v025i01.

Lu D, Chatterjee S, Xiao K, Riedel I, Huang C-K, Costa A, Cushman S, Neufeldt D, Rode L, Schmidt A, Juchem M, Leonardy J, Büchler G, Blume J, Gern O-L, Kalinke U, Wen Tan WL, Foo R, Vink A, van Laake LW, van der Meer P, Bär C & Thum T (2022) A circular RNA derived from the insulin receptor locus protects against doxorubicin-induced cardiotoxicity . *Eur. Heart J.* 43, 4496–4511. Available at: https://doi.org/10.1093/eurheartj/ehac337.

Maurício T, Aveiro S, Guedes S, Lopes D, Melo T, Neves BM, Domingues R & Domingues P (2022) Multi-Omic Profiling of Macrophages Treated with Phospholipids Containing Omega-3 and Omega-6 Fatty Acids Reveals Complex Immunomodulatory Adaptations at Protein, Lipid and Metabolic Levels. *Int. J. Mol. Sci.* 23.

Noberini R & Bonaldi T (2017) A Super-SILAC Strategy for the Accurate and Multiplexed Profiling of Histone Posttranslational Modifications. *Methods Enzymol.* 586, 311–332.

Noberini R, Savoia EO, Brandini S, Greco F, Marra F, Bertalot G, Pruneri G, McDonnell LA & Bonaldi T (2021) Spatial epi-proteomics enabled by histone post-translational modification analysis from low-abundance clinical samples. *Clin. Epigenetics* 13, 145. Available at: https://doi.org/10.1186/s13148-021-01120-7.

Nóbrega-Pereira S & de Jesus BB (2020) Cellular Reprogramming and Aging. , 73–91.

Pluskal T, Castillo S, Villar-Briones A & Orešič M (2010) MZmine 2: Modular framework for processing, visualizing, and analyzing mass spectrometry-based molecular profile data. *BMC Bioinformatics* 11, 395. Available at: https://doi.org/10.1186/1471-2105-11-395.

Subramanian A, Tamayo P, Mootha VK, Mukherjee S, Ebert BL, Gillette MA, Paulovich A, Pomeroy SL, Golub TR, Lander ES & Mesirov JP (2005) Gene set enrichment analysis: a knowledge-based approach for interpreting genome-wide expression profiles. *Proc. Natl. Acad. Sci. U. S. A.* 102, 15545–15550.

Tsugawa H, Ikeda K, Takahashi M, Satoh A, Mori Y, Uchino H, Okahashi N, Yamada Y, Tada I, Bonini P, Higashi Y, Okazaki Y, Zhou Z, Zhu Z-J, Koelmel J, Cajka T, Fiehn O, Saito K, Arita M & Arita M (2020) A lipidome atlas in MS-DIAL 4. *Nat. Biotechnol.* 38, 1159–1163.

Vizcaíno JA, Deutsch EW, Wang R, Csordas A, Reisinger F, Ríos D, Dianes JA, Sun Z, Farrah T, Bandeira N, Binz P-A, Xenarios I, Eisenacher M, Mayer G, Gatto L, Campos A, Chalkley RJ, Kraus H-J, Albar JP, Martinez-Bartolomé S, Apweiler R, Omenn GS, Martens L, Jones AR & Hermjakob H (2014) ProteomeXchange provides globally coordinated proteomics data submission and dissemination. *Nat. Biotechnol.* 32, 223–226.

Young MD, Wakefield MJ, Smyth GK & Oshlack A (2010) Gene ontology analysis for RNA-seq: accounting for selection bias. *Genome Biol.* 11, R14.

Yuan M, Breitkopf SB, Yang X & Asara JM (2012) A positive/negative ion–switching, targeted mass spectrometry–based metabolomics platform for bodily fluids, cells, and fresh and fixed tissue. *Nat. Protoc.* 7, 872–881. Available at: https://doi.org/10.1038/nprot.2012.024.

Yuan Z-F, Sidoli S, Marchione DM, Simithy J, Janssen KA, Szurgot MR & Garcia BA (2018) EpiProfile 2.0: A Computational Platform for Processing Epi-Proteomics Mass Spectrometry Data. *J. Proteome Res.* 17, 2533–2541.
